# Enhanced phosphorus recycling during past oceanic anoxia amplified by low rates of apatite authigenesis

**DOI:** 10.1126/sciadv.abn2370

**Published:** 2022-07-01

**Authors:** Nina M. Papadomanolaki, Wytze K. Lenstra, Mariette Wolthers, Caroline P. Slomp

**Affiliations:** Department of Earth Sciences, Faculty of Geosciences, Utrecht University, Utrecht, Netherlands.

## Abstract

Enhanced recycling of phosphorus as ocean deoxygenation expanded under past greenhouse climates contributed to widespread organic carbon burial and drawdown of atmospheric CO_2_. Redox-dependent phosphorus recycling was more efficient in such ancient anoxic marine environments, compared to modern anoxic settings, for reasons that remain unclear. Here, we show that low rates of apatite authigenesis in organic-rich sediments can explain the amplified phosphorus recycling in ancient settings as reflected in highly elevated ratios of organic carbon to total phosphorus. We argue that the low rates may be partly the result of the reduced saturation state of sediment porewaters with respect to apatite linked to ocean warming and acidification and/or a decreased availability of calcium carbonate, which acts as a template for apatite formation. Future changes in temperature and ocean biogeochemistry, induced by elevated atmospheric CO_2_, may similarly increase phosphorus availability and accelerate ocean deoxygenation and organic carbon burial.

## Teaser

Low rates of apatite authigenesis in anoxic, warm, CO_2_-rich oceans promoted widespread deoxygenation and organic carbon burial.

## INTRODUCTION

Phosphorus (P) is a key nutrient for marine phytoplankton and is the principal limiting nutrient on geological time scales (*1*). Through its control on primary productivity, P availability can affect organic carbon (C_ORG_) burial and atmospheric carbon dioxide (CO_2_) and oxygen (O_2_) (*2*). The availability of P in the surface ocean, in turn, is determined by riverine P input, P burial in sediments, and recycling of P (*3*). Sinking of organic matter is the main route of delivery of P and C_ORG_ to sediments (*3*). Recycling of P relative to C_ORG_ from sediments is enhanced upon ocean deoxygenation (*2*, *4*, *5*). Enhanced recycling of P under anoxic conditions is the combined result of increased preservation of C_ORG_ (*6*), preferential release of P from organic matter (*7*, *8*), including microbial polyphosphates (*5*), and less efficient retention of P in mineral form, as either iron (Fe) oxide–bound P (*5*) or authigenic apatite (*3*, *5*). Organic P (P_ORG_), Fe-bound P, authigenic apatite, and detrital P together make up the total P (P_TOT_) pool in marine sediments.

Changes in P recycling are reflected in elevated values of C_ORG_ over P_TOT_ (C_ORG_/P_TOT_), as observed in both modern and ancient marine sediments (*5*, *7*). The range of C_ORG_/P_TOT_ values for deposits formed during past oceanic anoxia is wider than that for modern sediments (*5*, *9*, *10*): While ancient values above 1000 mol mol^−1^ are not uncommon, the modern range does not exceed 400 mol mol^−1^ (*5*, *9*, *10*). Various explanations for these elevated C_ORG_/P_TOT_ values, which occur in both shallow and deep marine settings (*9*), have been proposed. For example, higher atmospheric CO_2_ may have increased the ratio of C_ORG_ to P_ORG_ (C_ORG_/P_ORG_) of phytoplankton (*10*). Because P_ORG_ is the primary source of P to sediments (*3*), and provides P for authigenic apatite formation, this would elevate C_ORG_/P_TOT_ values. Alternatively, lower atmospheric O_2_ in Earth’s past could have caused more reducing conditions near the seafloor (*5*) and an increased release of P from Fe oxides and organic matter, relative to modern anoxic systems. However, many modern anoxic systems are rich in hydrogen sulfide and hence contain negligible Fe oxide bound P (*11*). It is therefore unlikely that Fe-bound P contents would have been much different in ancient anoxic sediments. Furthermore, sediment C_ORG_/P_ORG_ values range up to at least 1200 in modern environments (*11*) and thus fall within the range of C_ORG_/P_TOT_ values for ancient anoxic environments (*9*). This suggests that, in these ancient sediments, a smaller proportion of the P released from organic matter and Fe oxides was retained in authigenic apatite. Thus, variations in apatite formation must have played a key role in modulating the response of C_ORG_/P_TOT_ to bottom water redox conditions. Rates of apatite formation are not directly redox-sensitive but depend heavily on the degree of porewater supersaturation with respect to apatite (*12*, *13*), besides depending on the presence of suitable templates that may include CaCO_3_ (*14*). Saturation depends on solute concentrations of the dominant components of the apatite mineral, notably phosphate (PO_4_) and calcium (Ca^2+^), as well as various environmental conditions such as temperature and pH (*12*).

Previous work has focused on C_ORG_/P_TOT_ trends in the geological record on the time scale of the whole Phanerozoic (*5*), but here, we focus on past ocean deoxygenation events associated with major changes in global biogeochemical cycles and climate. We specifically assess the impact of bottom water anoxia and apatite authigenesis on P recycling during three well-studied intervals: the Toarcian Oceanic Anoxic Event [T-OAE; 183 million years (Ma)], Oceanic Anoxic Event 2 (OAE2; 94 Ma), and Paleocene-Eocene Thermal Maximum (PETM; 56 Ma; table S1). T-OAE and OAE2 are two of the most severe ocean deoxygenation events in Earth’s history (*15*), with euxinic waters (no oxygen and presence of free sulfide) covering 2 to 5% of the seafloor (*16*–*18*) [modern: ~0.15% (*17*)]. Deoxygenation during PETM was widespread (*19*), but anoxia and euxinia were not very prevalent, as evident from the 1‰ sulfur isotope excursion (*20*) [T-OAE: 5 to 7‰ (*16*); OAE2: 2 to 6‰ (*17*)]. All three events were associated with high atmospheric CO_2_ (*15*, *21*), increased temperatures (*15*, *21*), lower than modern pH (*22*–*24*), and enhanced P recycling and C_ORG_ burial (*25*–*28*). In addition to our study of these three events, we also investigate three Mediterranean sapropel deposits (i-282c, S5, and S1), which allow us to assess enhanced P recycling under modern CO_2_ and O_2_ conditions (*5*, *29*). These sapropels provide a gradient in the persistence and vertical extent of water column euxinia: During formation of i-282c sapropel, hydrogen sulfide reached the photic zone (*30*), whereas only deeper parts of the water column were euxinic during sapropels S5 and S1 (*31*). Last, we include the Arabian Sea and Black Sea, two modern, low-O_2_ environments (with bottom water concentrations of <2.6 μM O_2_ and 418 μM H_2_S, respectively, at the time of sampling of the sites relevant to this study) where apatite formation is known to occur (*11*, *32*).

For each deposit, we compile and compare sediment values of C_ORG_/P_TOT_, C_ORG_/P_ORG_ (due to diagenesis, only for sapropels and modern sediments), and Fe over aluminum (Fe/Al) and molybdenum (Mo), two redox proxies (*33*, *34*), to illustrate that anoxia alone does not explain the high C_ORG_/P_TOT_ values of ancient sediments. Using reactive transport simulations and saturation state calculations for apatite formation in organic-rich deep-sea sediments, we show that the combination of enhanced redox-dependent recycling of P from organic matter and low rates of apatite formation can explain the high C_ORG_/P_TOT_ values observed in ancient anoxic marine settings.

## RESULTS AND DISCUSSION

### Redox-dependent P recycling

**Fig. 1.**
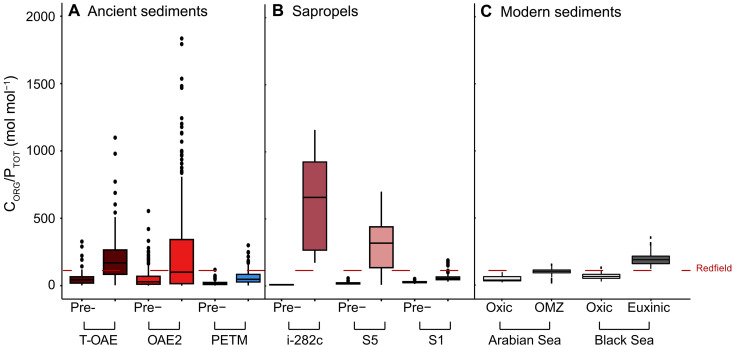
Past and present CORG/PTOT values. Compilation of C_ORG_/P_TOT_ values for ancient sediments (**A**), sapropels (**B**), and modern sediments (**C**). Ancient sediments were deposited during T-OAE, OAE2, and PETM. The three Eastern Mediterranean sapropels are i-282c, S5, and S1; pre-event C_ORG_/P_TOT_ values are presented separately. Modern sediments were deposited in and below the Arabian Sea OMZ, on the oxic Black Sea shelf, and in the adjacent euxinic basin. Horizontal lines within the boxplots represent median values. The horizontal red line indicates the Redfield C_ORG_/P_ORG_ value of 106:1 (*36*).

Median C_ORG_/P_TOT_ values for all ancient sediments and sapropels are elevated compared to values preceding the onset of widespread deoxygenation (Fig. 1, A and B), in accordance with a transition toward enhanced P recycling. Similarly, median C_ORG_/P_TOT_ values are higher in the modern Arabian Sea oxygen minimum zone (OMZ) and euxinic Black Sea basin, when compared to values for their respective oxic sediments (Fig. 1C), in accordance with redox-dependent P recycling. Median values for T-OAE, i-282c, and S5 (169, 655, and 315, respectively), and maximum C_ORG_/P_TOT_ values for all ancient sediments, exceed the Redfield ratio of 106 mol mol^−1^ (*35*), indicating sediment deposition in anoxic bottom waters (Fig. 1, A and B, and table S2) (*6*). Higher than modern C_ORG_/P_TOT_ values are observed for T-OAE, OAE2, and i-282c and S5 sapropels. These values are not found during PETM and for sapropel S1. The highest maxima, in excess of 1000 mol mol^−1^, are observed for T-OAE, OAE2, and i-282c sediments (1099, 1835, and 1156, respectively). The lowest median values, and smallest spread, are observed for PETM and sapropel S1 (40 and 53, respectively). This agrees with earlier work demonstrating that anoxia and euxinia were overall more prevalent during T-OAE, OAE2, and i-282c and S5 sapropels, when compared to PETM and sapropel S1 (*15*, *30*, *36*). Median values of C_ORG_/P_TOT_ for the Arabian Sea OMZ and the euxinic Black Sea are 103 and 189 mol mol^−1^, respectively, and thus fall within the range of median values for the paleoenvironments. However, the overall range in C_ORG_/P_TOT_ values in modern sediments from anoxic settings is much lower than during the paleo-events and remains below 400, as observed previously (*5*).

**Fig. 2.**
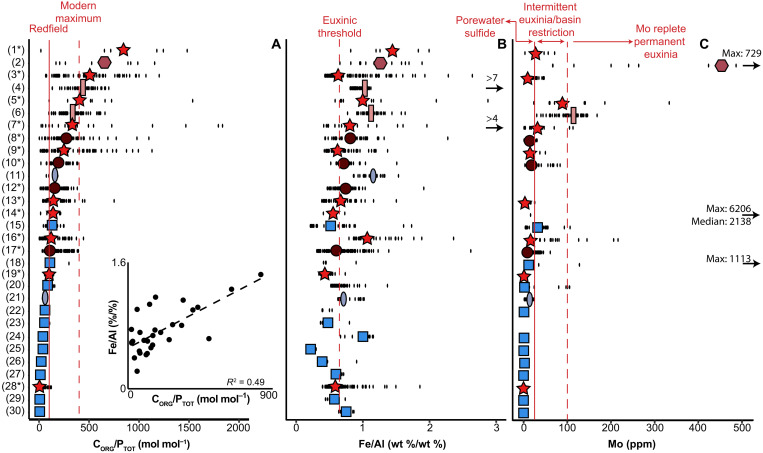
Redox proxy compilation. Site-specific compilation of C_ORG_/P_TOT_ (**A**), Fe/Al (**B**), and Mo (**C**) values for T-OAE (circle), OAE2 (star), PETM (square), and i-282c (rhombus), S5 (rectangle), and S1 (oval) sapropels. Colors are additional indicators of the corresponding events. Two samples with high Fe/Al values of 4 and 7 weight % (wt %)/wt % are indicated in the right-hand panel with arrows. Similarly, the maximum and median values exceeding 500 ppm are indicated with arrows in (C). The vertical lines in (A) indicate the Redfield C_ORG_/P_ORG_ value (solid) and the modern maximum C_ORG_/P_TOT_ value of 400 [dashed; (*5*)]; in (B), the Fe/Al threshold for sediments overlain by euxinic bottom waters [dashed; (*33*)]; in (C), the 25-ppm threshold for intermittent euxinia or permanent euxinia with basinal restriction (solid) and the 100-ppm threshold for permanent euxinia under Mo replete conditions [dashed; (*34*)]. For (C), T-OAE and OAE2 site values are likely affected by basinal restriction (asterisks). The figure inset depicts the correlation between the median values of the C_ORG_/P_TOT_ and Fe/Al values for each site. The black dashed line is the linear regression line (*R*^2^ = 0.47). The sites are sorted from highest median C_ORG_/P_TOT_ to lowest. The site names corresponding to each number are given in table S3.

A comparison of C_ORG_/P_TOT_ values with the corresponding Fe/Al for each study site shows that ancient sediments with median C_ORG_/P_TOT_ > 106 are generally characterized by median values of Fe/Al > 0.66, indicating anoxic and euxinic facies (Fig. 2, A and B, and table S3) (*33*). Most of the data points (~70%) with both Fe/Al > 0.66 and C_ORG_/P_TOT_ > 106 correspond to sediments from T-OAE, OAE2, i-282c, and S5. This indicates that, generally, C_ORG_/P_TOT_ values are higher under more reducing conditions and supports the utility of C_ORG_/P_TOT_ values as a redox proxy. Median Mo concentrations (Fig. 2C) are generally below 25 parts per million (ppm), signifying non-euxinic conditions with sulfide restricted to pore waters (*34*). Median Mo values above 25 ppm mostly correspond to median C_ORG_/P_TOT_ > 106. Mo values above 25 and/or 100 ppm do not correspond to specific C_ORG_/P_TOT_ median or maximum values. Notably, many of the ancient sediments with high C_ORG_/P_TOT_ values are also very rich in organic matter and frequently have a C_ORG_ content of 10 to 20% (*9*, *25*, *37*–*39*).

We note that enhanced recycling of P from organic matter under low [O_2_] should also be reflected in a high C_ORG_/P_ORG_. Unfortunately, however, the application of P speciation is limited by sample storage (*40*) and sediment age (*39*). As degradation of organic matter continues during burial, sediment P_ORG_ values gradually decrease on time scales of thousand years (ka) to million years (Ma). This can result in high C_ORG_/P_ORG_ values that do not reflect the original conditions during deposition (*39*). The P that is released from organic matter during long-term burial is typically retained as authigenic apatite (*40*); hence, C_ORG_/P_TOT_ values are not affected. This long-term diagenetic sink switching precludes the use of P speciation data for T-OAE, OAE2, and PETM. The age of sapropel S5, however, places it in the 600- to 700-ka window in which C_ORG_/P_ORG_ values for the Mediterranean Sea are thought to mostly reflect depositional conditions (*39*). For sapropel i-282c, C_ORG_/P_ORG_ values could be somewhat affected by diagenesis. Elevated C_ORG_/P_ORG_ values are a prerequisite for elevated C_ORG_/P_TOT_ values, which are additionally modulated by authigenic apatite formation, as discussed in the next section.

### Formation of apatite as a modulator of sediment C_ORG_/P_TOT_

**Fig. 3.**
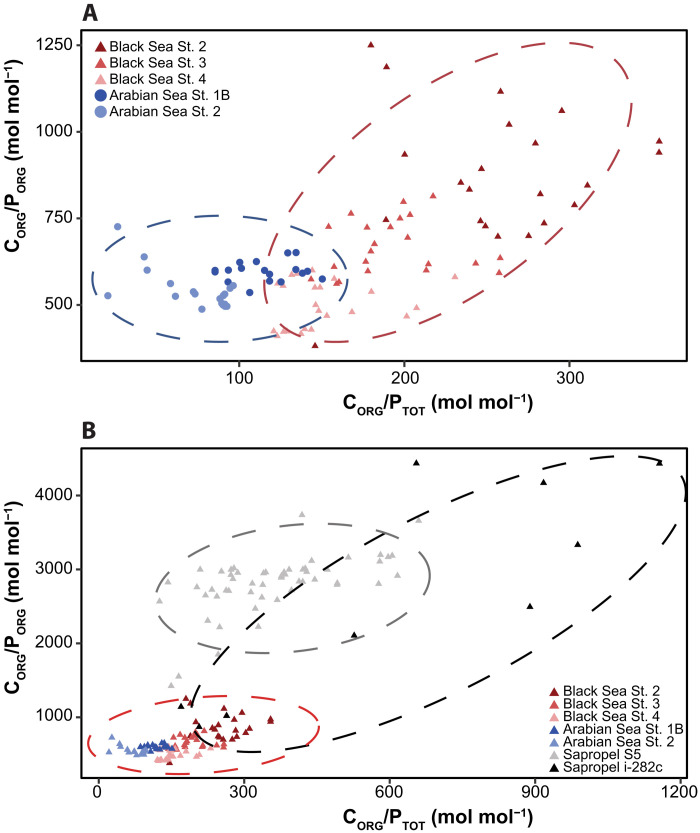
Crossplots for sediment CORG/PTOT and CORG/PORG. Relationship between sediment C_ORG_/P_TOT_ and C_ORG_/P_ORG_ for (**A**) three sites in the euxinic basin of the Black Sea (*11*) and two sites from the Arabian Sea OMZ (*32*), and (**B**) the Black Sea and Arabian Sea data combined with data for i-282c (*38*) and S5 sapropels (*39*). Dashed circles indicate the approximate extent of values for each area or event, as well as the general trend.

In sediments of the modern Arabian OMZ, C_ORG_/P_ORG_ values exceed 500 mol/mol but C_ORG_/P_TOT_ values are <200 mol mol^−1^ (Fig. 3) (*32*). Similarly, in sediments of the euxinic Black Sea basin, maximum C_ORG_/P_ORG_ values are well above 1000 mol mol^−1^, but C_ORG_/P_TOT_ does not exceed 400 mol mol^−1^ (*11*). These differences in C_ORG_/P_ORG_ and C_ORG_/P_TOT_ values in the Arabian Sea and Black Sea are in accordance with the differences in bottom water redox conditions (anoxic versus euxinic), lower P release from organic matter, and higher rates of apatite formation in the former system (*11*, *32*). Values of C_ORG_/P_ORG_ and C_ORG_/P_TOT_ values for the two sapropels with the most reducing conditions [i-282c (*38*) and S5 (*39*)], in contrast, range up to nearly 4300 and 1200 mol mol^−1^, respectively, and hence are at least an order of magnitude higher than those for the Arabian Sea and Black Sea. If apatite formation was capable of retaining the P released from organic matter, C_ORG_/P_TOT_ values for the sapropels should not have exceeded the modern maximum.

To further assess the potential impact of variations in the rate of apatite authigenesis on C_ORG_/P_TOT_ ratios, we performed sensitivity analyses with a reaction transport model describing biogeochemical processes in Arabian Sea sediment (*32*). The model settings for a site directly below the OMZ, as published previously, were modified to capture the environmental characteristics of a deep-sea sediment overlain by anoxic bottom waters (table S7). Important changes to the model include setting bottom water oxygen and the rates of bioturbation and bioirrigation to zero, and increasing the flux of organic matter to the sediment-water interface. We modeled the rate of authigenic apatite formation in a simplified manner as the product of a rate constant (*k*_17_) and the dissolved PO_4_ concentration (*32*).

**Fig. 4.**
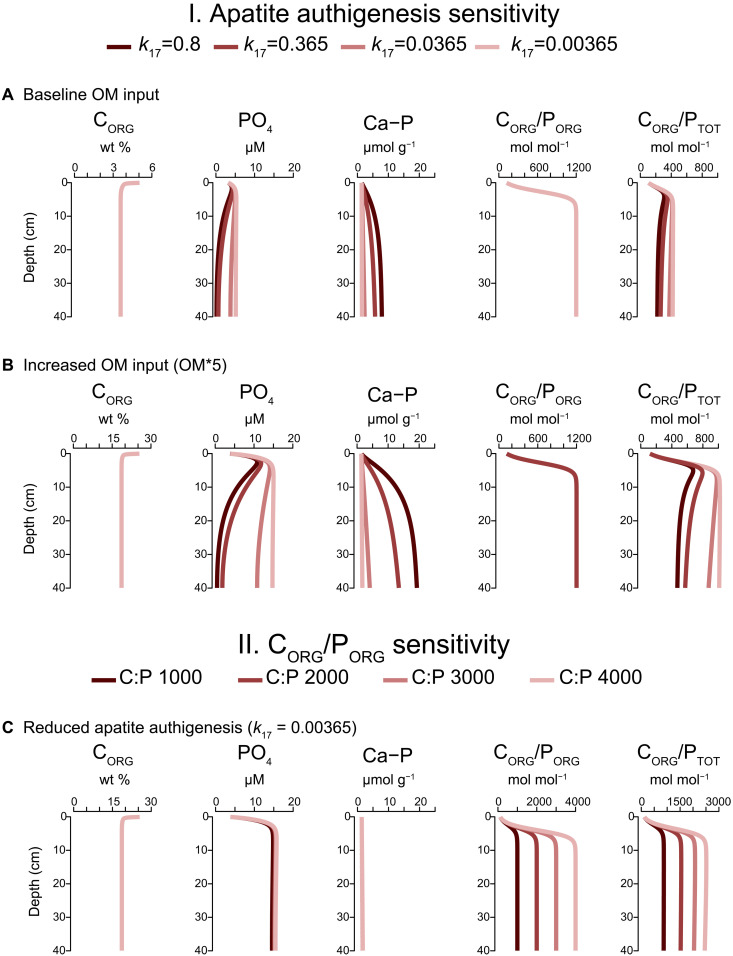
Reactive transport model response to variations in apatite authigenesis and CORG/PORG values. Depth profiles of C_ORG_, porewater PO_4_, detrital and authigenic apatite (Ca-P), C_ORG_/P_ORG_, and C_ORG_/P_TOT_ as calculated with a reactive transport model in scenarios with variations in the rate of apatite authigenesis with (**A**) a baseline input of organic matter and (**B**) a fivefold higher organic matter (OM) input and (**C**) scenarios with variations in the value of C_ORG_/P_ORG_ for the OM^γ^ fraction and a low rate of apatite authigenesis. Rates of authigenesis were modified by altering *k*_17_ values. For further details on the model and the simulations, see Materials and Methods and the Supplementary Materials (tables S4 to S6).

We first assessed the effect of variations in the value of the rate constant *k*_17_ on C_ORG_/P_TOT_ values for a moderate rate of organic matter input (Fig. 4A). The maximum C_ORG_/P_ORG_ ratios were forced to a value of ~1200 mol mol^−1^ by imposing an enhanced release of dissolved PO_4_ upon organic matter degradation, to allow full focus on the effects of variations in authigenic apatite formation on C_ORG_/P_TOT_ values. The results show that lower rates of apatite authigenesis correspond to high C_ORG_/P_TOT_ ratios. In these scenarios, C_ORG_/P_TOT_ ratios only exceed the maximum modern value of 400 mol mol^−1^ in a scenario with near negligible apatite formation. In a second set of scenarios, we impose a fivefold increase in the input of organic matter (Fig. 4B). The higher production of PO_4_ in the porewater when compared to the previous scenarios leads to more apatite formation for the same values of *k*_17_ but higher C_ORG_/P_TOT_ values, in this case up to ~1000 mol mol^−1^. Hence, when rates of organic matter input are high and rates of authigenic P formation are low, P retention in apatite becomes so inefficient that the high C_ORG_/P_TOT_ values typical for ancient sediments from anoxic basins are observed. Even higher ratios of C_ORG_/P_TOT_ can be achieved when allowing for C_ORG_/P_ORG_ ratios up to 4000 (Fig. 4C), emphasizing the critical role of both low rates of apatite formation and high C_ORG_/P_ORG_ in elevating C_ORG_/P_TOT_ values.

### Causes of low rates of apatite authigenesis

In situ mechanisms of apatite formation in marine sediments are difficult to resolve because of the multitude of biogeochemical processes and the various potential templates involved, and slow rates of formation (*3*). A higher degree of supersaturation of the porewater with respect to apatite will, in principle, promote mineral formation (*12*, *13*). Carbonate fluorapatite (CFA) is the most common type of authigenic apatite in marine sediments, and its rate of formation (*r*_CFA_) can be expressed as (*41*)

$$r_{\mathrm{CFA}}=k_{\mathrm{CFA}}(\Omega_{\mathrm{CFA}}-1)$$ (1)

where *k*_CFA_ is a rate constant and Ω_CFA_ is the saturation state with respect to CFA (with supersaturation when Ω_CFA_ > 1). Potential controls on the saturation state include porewater [PO_4_], fluoride ([F^−^]) (*42*, *43*), and [Ca^2+^] (*41*). This formula suggests that the growth rate of CFA is nearly linearly dependent on the saturation state, but this is likely an oversimplification. For fluorapatite, for example, the rate of crystal growth can accelerate above a certain threshold saturation state (*44*). It is therefore likely that the rate of formation of CFA also increases more strongly at higher degrees of supersaturation, especially in the presence of suitable nucleation surfaces (*14*), which can be provided by calcium carbonate (CaCO_3_), microbially derived organic matter (*14*, *45*, *46*), and/or polyphosphates (*5*, *47*). In persistently anoxic settings, a role for microbial polyphosphate as a template may, however, be excluded (*5*). Last, laboratory experiments have revealed a strong sensitivity of CFA solubility to temperature and pH (*12*).

**Fig. 5.**
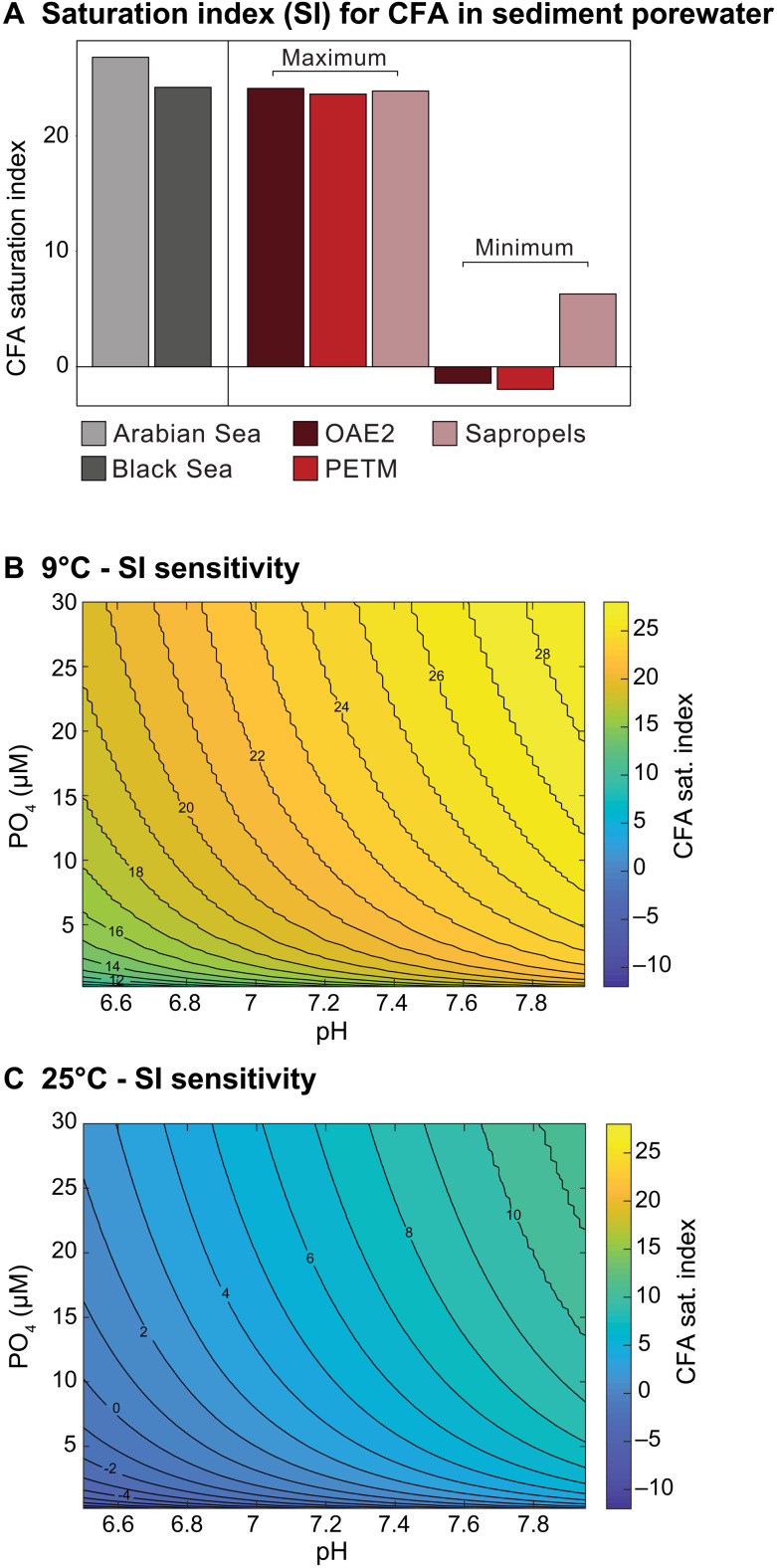
CFA saturation index values and sensitivity to temperature, pH and [PO_4_]. SIs for CFA as calculated with PHREEQC for (**A**) the modern Arabian Sea and Black Sea anoxic sediments and estimated for OAE2, PETM, and eastern Mediterranean sapropels and for the Arabian Sea OMZ at 9°C (**B**) and 25°C (**C**) for a range of [PO_4_] (0.2 to 30 μM) and pH (6.5 to 7.95) values. Input values for the simulations are given in table S9.

Here, we use PHREEQC to calculate the response of porewater saturation states, with respect to CFA, at our study sites to variations in pH, temperature, and porewater chemistry (Supplementary Text and table S9). For modern Arabian Sea OMZ and euxinic Black Sea surface sediments, where CFA formation is observed (*11*, *32*), we find an average saturation index (SI) of ca. 24 and 27, respectively, indicating supersaturation of the porewaters with respect to CFA (Fig. 5A and Supplementary Text). A subsequent sensitivity analysis for porewaters of the anoxic Arabian Sea OMZ Station 1B reveals that an increase in temperature and a decrease in pH and [PO_4_] result in SI reduction. Within the tested ranges (*T*: 9° to 25°C; pH: 6.5 to 7.95; [PO_4_]: 0.2 to 30 μM), temperature has the largest impact on the SI (Fig. 5, B and C). For any given pH and [PO_4_] combination, the SI at 9°C is 17 units higher than at 25°C, whereas the difference in SI is ~9.5 units between the lowest and highest pH, and ~11 units between the highest and lowest [PO_4_]. Notably, above a [PO_4_] of ca. 5 μM, the changes in SI for further increases in [PO_4_] are relatively small when compared to a change in pH. Undersaturation is achieved at 25°C (Fig. 3C), when [PO_4_] < 10 μM and for pH < 7.8.

Increasing evidence demonstrates that many of our ancient sediments, characterized by persistent anoxia or euxinia (table S11), and high C_ORG_/P_TOT_ (Figs. 1 and 2), were deposited under higher than modern temperatures and lower pH. During OAE2, surface waters warmed to 35° to 36°C (*48*), while deep water temperatures were likely in the 12° to 24°C range (*49*). As a result, most OAE2 sites in our data compilation (Fig. 2), including those located at great depths (>2000 m) (*37*), probably experienced temperatures in excess of 20°C. Similarly, during T-OAE, the European Shelf, where our study sites were located, was subject to substantial warming (*50*, *51*), with bottom temperatures in the western Tethys increasing by 3.5°C and exceeding 20°C (*52*). Last, the deposition of i-282c and S5 sapropels likely coincided with warming of bottom waters in the Eastern Mediterranean (*53*, *54*), exceeding the modern-day temperature of ~14°C (*55*). Constraints on pH are more uncertain, but evidence suggests an increase in ocean pH from ~7.5 to 8.2 over the last ca. 100 Ma (*23*) and a decrease in oceanic pH due to ocean acidification during T-OAE (*22*) and PETM (*24*). Furthermore, pH values in modern surface sediments frequently range between 6.9 and 8.0, and are generally lower than the pH of the overlying waters (*56*). Our calculations point toward supersaturation of porewaters with respect to CFA for OAE2, PETM, and sapropel sediments at low temperatures (14°C) and maximum pH and PO_4_ values (Fig. 5A and table S9). Conversely, when assuming warming to 25°C (17°C for the Eastern Mediterranean), a pH of 6.9, and [PO_4_] of 1 μM, the SI values markedly decrease, indicating undersaturation with respect to CFA for OAE2 and PETM. These conditions would likely have hindered increased CFA authigenesis, which would have had a larger impact on global sediment P recycling during OAE2 than PETM because of the larger area of anoxic (and frequently sulfidic) bottom waters during the former event (*15*, *57*). The effects of changes in alkalinity and major ions (e.g., [Ca^2+^] and [Mg^2+^]) were negligible (Supplementary Text). Notably, the thermodynamic effects of temperature and pH on the solubility of CFA can be partially offset by kinetic effects acting in the opposite direction, increasing the rate constant for CFA formation (*44*). However, the compound result of the kinetic effects on the rate of CFA formation (Eq. 1) related to temperature and pH changes, expected for OAE2 and PETM [at most an increase by a factor of ~4.5; table S9; (*44*)], does not compensate for the effect of the decrease in SI (table S9) and hence Ω_CFA_ on the rate of CFA formation. The kinetic effects become irrelevant when undersaturation is reached and CFA formation comes to a halt.

The presence of calcium carbonate (CaCO_3_) in sediments affects the potential for CFA authigenesis, by providing suitable nucleation surfaces (*14*). Calcium carbonate contents for the most anoxic ancient sediments (T-OAE, OAE2, i-282c, and S5) are generally much lower than for modern Arabian and Black Sea sediments, with average values for the two OAEs and i-282c below 20% at most sites (table S10). In addition, an inverse correlation between sediment CaCO_3_ contents and C_ORG_/P_TOT_ ratios is observed for i-282c (fig. S1) (*38*), similar to S5 (*39*). Low sediment CaCO_3_ was the consequence of reduced CaCO_3_ input from overlying waters, as high *P*co_2_ (partial pressure of CO_2_) and oceanic anoxia affected phytoplankton species (*58*), and CaCO_3_ dissolution upon a decreased pH (*22*–*24*). Consequently, the low CaCO_3_ content at euxinic sites during the T-OAE, OAE2, and sapropel formation may have hindered an increase in CFA authigenesis, rendering it insufficient to retain the increased P supply to sediments.

### Oceanic impact of enhanced P recycling

While temperature, pH, and template availability can control the rate of apatite authigenesis, low rates of apatite formation only led to extremely high C_ORG_/P_TOT_ values in organic-rich sediments experiencing anoxia and, probably, euxinia, when overall recycling of P is enhanced. Under oxic conditions, when P is retained in Fe oxides and in organic matter, and C_ORG_/P_ORG_ values remain close to the Redfield ratio of 106:1, C_ORG_/P_TOT_ will remain low regardless of whether rates of apatite formation are low. Therefore, as sites during PETM and sapropel S1 were generally hypoxic and/or not euxinic (table S11), and anoxic/euxinic conditions were less widespread for these two time intervals (*15*, *31*), C_ORG_/P_TOT_ ratios remained lower compared to T-OAE, OAE2, and i-282c and S5 sapropels (Figs. 1 and 2).

To further illustrate the effect of increased P recycling from organic matter on P retention and C_ORG_ burial, we use two versions of a one-box biogeochemical model for the oceanic C and P cycles (4): the original model in which apatite formation is related to the rate of organic matter decay and a model version that assumes a lower rate of apatite formation accompanying increased anoxia [expressed as the degree of anoxia (DOA)]. Deoxygenation is generated by decreasing the rate of ocean overturning (*4*).

**Fig. 6.**
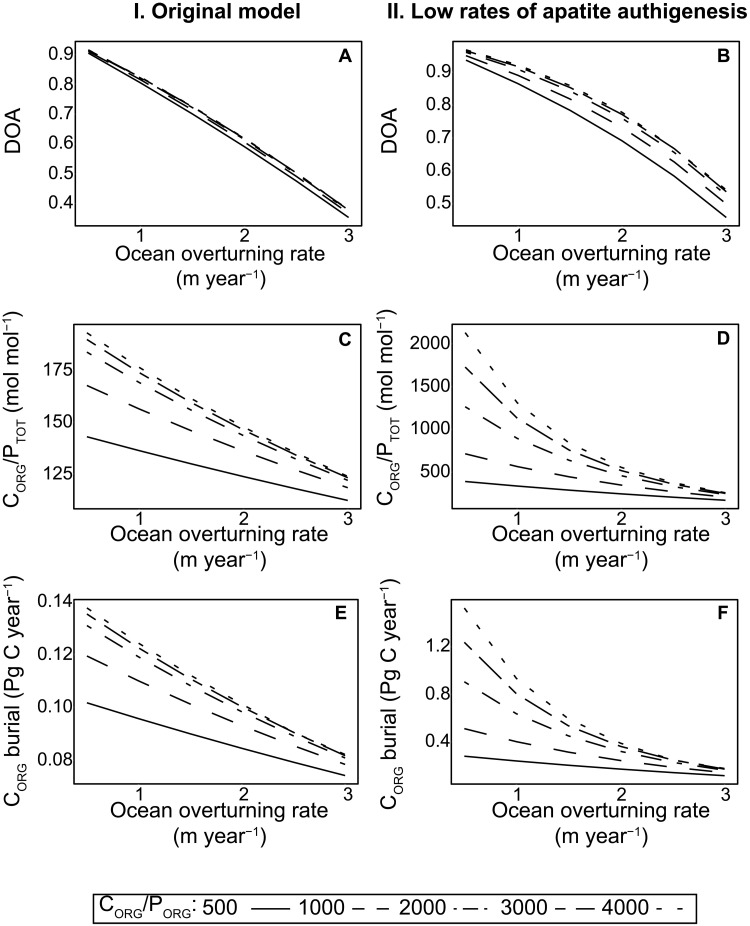
Box model output for simulations using the original (*4*) model (I) and with low rates of apatite authigenesis (II). The Degree of Anoxia (DOA; **A** and **B**), C_ORG_/P_TOT_ ratio (**C** and **D**) and C_ORG_ burial rate (**E** and **F**) are shown for increasing ocean overturning rates and maximum C_ORG_/P_ORG_ (lines). Results are shown for ocean overturning rates in the range of 0.5 to 3 m year^−1^ and C_ORG_/P_ORG_ of 500 to 4000 mol mol^−1^.

In steady-state simulations for an oxic ocean (low values of DOA; Fig. 6, A and D), C_ORG_/P_TOT_ values always remain within the modern range (Fig. 6, B and E). Under increasing deoxygenation (high values of DOA), only simulations with high maximum C_ORG_/P_ORG_ values and low rates of authigenic apatite burial capture the range of C_ORG_/P_TOT_ values for ancient sediments (maximum 2000 mol mol^−1^; Fig. 4E). When authigenic P burial is not low, C_ORG_/P_TOT_ values remain in the lower half of the modern range (<200 mol mol^−1^). In our model simulations for an ocean that is largely anoxic, low rates of authigenic apatite formation result in an almost 10-fold increase in the burial rate of C_ORG_ (Fig. 6, C and F).

In conclusion, our data compilation and modeling indicate that low and/or reduced rates of apatite authigenesis are a key factor that can explain the higher than modern recycling of P from ancient sediments, which is reflected in high C_ORG_/P_TOT_ values. Under anoxic conditions, with high organic matter input and P regeneration, low authigenesis rates result in increased C_ORG_/P_TOT_ values and enhanced C_ORG_ burial. A low saturation state, or even undersaturation, with respect to apatite could have facilitated these circumstances. Our results show that higher ocean temperatures, lower porewater pH, and low(er) CaCO_3_ contents could have hampered apatite formation and P retention in the sediment during past periods of oceanic anoxia. While additional factors could have played a role at individual sites, the mechanism we propose can have a global impact and modify the previous model under which redox was the sole control of high ancient C_ORG_/P_TOT_ values. The lack of high rates of apatite formation during sapropel formation (*36*), in particular, is a warning that much faster recycling of P relative to C_ORG_ is possible in the ocean under modern climate change. Areas, such as parts of the deep ocean, which currently have low organic matter input rates and do not experience apatite authigenesis, may be especially vulnerable in the future. Increased P regeneration could contribute to a further acceleration of ocean deoxygenation by stimulating primary productivity (*5*), C_ORG_ burial, and other key feedbacks in the global carbon and oxygen cycles.

## MATERIALS AND METHODS

### Data compilation

#### Event selection

The aim of this study is to elucidate what mechanisms led to stronger P recycling in ancient anoxic marine environments when compared to modern low oxygen settings. For this purpose, we selected intervals from the geological record that varied strongly in their degree of deoxygenation, sediment composition, and climatic conditions during deposition. Specifically, we use data for sediments from T-OAE (183 Ma ago), OAE2 (94 Ma ago), and PETM (56 Ma ago), as well as for three Mediterranean sapropels (i-282c, S5, and S1) and two modern anoxic marine areas: the Arabian Sea and the Black Sea. The ancient and sapropel data cover sediments deposited before and during the spread of deoxygenation. For the Arabian Sea, we focus on sediments from within and below the OMZ, while the Black Sea data include sediments from the oxic shelf and the euxinic deep basin. The ancient and modern sediments cover both shallow and deep depositional settings (table S1). The full list of references for the data used in this study is given per site in table S1 and at the end of this document.

T-OAE and OAE2 are two of the most severe deoxygenation events in Earth’s history (*15*). Sulfur isotope values increased by 5 to 7‰ for T-OAE (*16*) and up to 5‰ for OAE2 (*17*). This increase was interpreted as a spread of euxinic conditions over 2 to 5% of the ocean seafloor, close to the estimate of ≤2% based on Mo isotopes (*18*). Uranium (U) isotopes for OAE2 suggest that 8 to 15% of the seafloor was anoxic (*59*). These conditions were caused by enhanced volcanic CO_2_ and methane emissions, which resulted in high atmospheric *P*co_2_ and a warmer and, in some areas, wetter climate (*15*). The greenhouse climate resulted in water column stratification, while increased continental weathering also led to an increase in nutrient supply to the oceans (*15*). The combination of these factors stimulated the spread of oceanic anoxia (*15*). Most of the known locations that were characterized by anoxic bottom waters during T-OAE were shallow and epicontinental (*25*). The sulfur isotope record, however, suggests a larger areal extent of anoxia (*16*), potentially including deep sites. Anoxic bottom waters occurred in both shallow and deep settings during OAE2 (*9*).

During PETM, similar perturbations of the exogenic C cycle resulted in widespread deoxygenation (*19*); however, anoxia and euxinia were less widespread in comparison to the two OAEs. The sulfur isotope excursion was 1‰ (*20*), suggesting a minor expansion in sulfidic bottom water area, and Mo isotopes also support less-extensive euxinia during PETM than during the two OAEs (*60*). In addition, U isotopes indicate that less than 2% of the seafloor was overlain by anoxic bottom waters (*61*). Regardless, it is hypothesized that the sediment burial of C_ORG_ resulted in CO_2_ drawdown during all three events (*25*, *62*, *63*).

In contrast to the large spatial extent of the anoxia during the three chosen events, the conditions that led to sapropel formation were restricted mostly to the Eastern Mediterranean. Intense rainfall during stronger monsoon intervals resulted in stratification and deoxygenation, which, in turn, increased the burial of C_ORG_ and the recycling of P (*38*, *64*). Bottom water conditions during the formation of S1 (10 ka ago) were generally less reducing than for S5 (125 ka ago) and i-282c (2.943 Ma ago) (*36*, *38*).

Other examples of intervals that experienced deoxygenation are the Late Permian, OAE1a, and the glacial-interglacial cycles. However, various indicators, such as the lack of high C_ORG_/P_TOT_, suggest that anoxia and euxinia were likely not as widespread, and P recycling was not as enhanced, as during T-OAE and OAE2 (*65*–*67*). These events are therefore not included in this study, as PETM and sapropel S1 already provide examples for milder deoxygenation and P recycling. In addition, there are more data available on the degree and extent of these changes for PETM (*19*).

#### Site selection

The sites for which data are presented here cover multiple depositional environments. The selection of sites is largely determined by the availability of data. For T-OAE, only shallow sites from the European Epicontinental Seaway are available, due to the destruction of oceanic crust during subduction. The four sites that we use are Schandelah, Dotternhausen, Yorkshite, and Rietheim. For OAE2, we compiled data for intermediate and deep ocean sites from the North Atlantic [DSDP (Deep Sea Drilling Project) 367, 386, 603, and 641 and ODP (Ocean Drilling Project) 1260 and 1276] as well as shallow shelf sites (Tarfaya, Bass River, and Wunstorf). The PETM sites cover mostly shelf and slope environments (Forada, Bass River, IB10, ODP 752, 959, and 1172, [IODP (International Ocean Discovery Project) M006] with one deep-sea site (IODP 1403). Sapropels were mostly deposited in the Eastern Mediterranean, and we compiled data for deeper sites (S1: MS21PC and KC19C; S5: PS25PC and KC19C; i-282c: ODP 969).

The Arabian Sea, in the northwestern Indian Ocean, is characterized by an OMZ (O_2_ < 20 μM) extending from ~200- to 1000-m depth. Stations 1B and 2 are located within the OMZ. The remaining stations (3 to 5, 6B, and 7 to 10) are located below the OMZ and characterized in this study as the oxic sites. The Black Sea is the largest strongly stratified marine basin in the world, with sulfidic bottom waters (<~100 m) (*68*). Stations 2 to 4 are located within the sulfidic basin, with station 2 being the deepest and most sulfidic. Stations 6A, 6B, and 7 are located on the oxic shelf. All references for the data used in this study can be found in the supplementary tables.

#### Proxy selection

In modern marine sediments, C_ORG_/P_ORG_ and C_ORG_/P_TOT_ ratios track bottom water redox conditions (*69*). A low [O_2_] favors the preservation of C_ORG_ while promoting the preferential regeneration of P_ORG_ (*7*). This results in elevated C_ORG_ contents relative to P_ORG_ and an increased C_ORG_/P_ORG_ ratio (*7*). The rate of authigenic mineral formation determines what proportion of the P released from organic matter is retained in the sediment (in the absence of Fe oxides). Sediment P speciation can provide insight into the response of different P sinks to environmental conditions during deposition (*3*).

For i-282c and S1 sapropels, apatite (both authigenic and biogenic) makes up at least a third of P_TOT_ burial (*38*, *70*). A large proportion of this authigenic apatite is thought to be a background flux from dust (*38*, *71*). During formation of S5 sapropel, authigenic apatite decreased and recycling of P to the water column increased (*39*). In the Arabian Sea, in contrast, the down-core increase of apatite at the most anoxic sites (1B and 2) indicates apatite authigenesis (*32*). This process also occurs in the deep euxinic basin of the Black Sea, where it begins in the upper centimeters of the sediment column (*11*), as it does in other areas in the modern ocean (*42*, *71*). We compare C_ORG_/P_ORG_ and C_ORG_/P_TOT_ ratios for S5 and i-282c sapropels, both of which have C_ORG_/P_TOT_ > 400 mol mol^−1^, to those in the modern Black Sea and Arabian Sea below. Data for C_ORG_/P_TOT_ and C_ORG_/P_ORG_, as shown in Figs. 1 and 3, are given in table S2.

Various proxies are used for the reconstruction of global and local bottom water redox conditions. While global proxies are useful for a comparison of the spatial extent of anoxic waters at the global scale between events (as discussed above), local redox information is required to evaluate whether differences in redox conditions can explain differences in C_ORG_/P_TOT_ values between sites. To assess the local occurrence and intensity of bottom water anoxia, three proxies are often used: sediment Mo, U, and Fe/Al ratio. As U data are only sparsely available, we elected to use the Fe/Al ratio and Mo proxies. High Fe/Al (>0.66 on a weight basis) is assumed to reflect enhanced input of Fe and its sequestration as pyrite in sediments under anoxic and sulfidic bottom waters (*33*). Mo concentrations can be used as an indicator for the presence of hydrogen sulfide, either in pore waters only or in the water column (*34*). Specifically, concentrations above the crustal average (1 to 2 ppm) and below ~25 ppm indicate a non-euxinic water column, with hydrogen sulfide restricted to pore waters. High values, exceeding 100 ppm, indicate that the water column was permanently euxinic. Intermediate values (25 to 100 ppm) can be the result of multiple factors such as intermittent water column euxinia, high sedimentation rates, or basinal restriction, leading to depleted Mo values in the water column. In this study, the latter factor is of importance specifically for T-OAE and OAE2 as both these events experience basinal restriction (*37*, *72*, *73*). As a result, values for permanently euxinic sites from these two events may not exceed the 100-ppm threshold.

### Reactive transport modeling

#### Model description

To investigate the mechanisms that control the formation of authigenic apatite and its impact on the C_ORG_/P_TOT_ ratio, a reactive transport model was applied. The model describes the mass balance of nine dissolved and seven particulate species (table S4) and is a modified version of that of (*74*), based on (*75*). The model domain consists of a one-dimensional grid of 400 evenly distributed cells that captures the interval from the sediment-water interface to a depth of 40 cm. All chemical species are subject to biogeochemical reactions, which are divided into primary redox and other biogeochemical reactions (table S5). The succession of oxidants during organic matter degradation (*76*) is described by means of Monod kinetics (table S6) (*77*). A new addition to the model is that enhanced regeneration of P from organic matter is modeled by assuming conversion of the β fraction of organic matter, with a Redfield C_ORG_/P_ORG_ ratio, to a refractory γ fraction with a higher C_ORG_/P_ORG_ ratio and release of the associated PO_4_ to the porewater (R24; table S5). Solids and solutes are transported by sediment accumulation. Solutes are additionally transported by molecular diffusion (*75*, *77*). Environmental parameters and reaction constants were mostly taken from Arabian Sea station 4 in (*32*), located below the OMZ, with several modifications to make the setting representative for a fully anoxic deep-sea environment (tables S7 and S8). Zero gradient boundary conditions were applied to the base of the model domain for all chemical species. The model was run to steady state for 25,000 years with a time resolution of 10 years.

#### Sensitivity analysis

A model sensitivity analysis was performed to investigate the impact of (i) changes in the kinetics of apatite formation, (ii) a higher organic matter input, and (iii) variations in the C_ORG_/P_ORG_ ratio of the refractory organic matter fraction. In the first set of simulations, the rate constant *k*_17_ for apatite formation was varied between four values: A high value for very high rates of authigenesis (0.8), the baseline value from (*32*) (0.365), and a factor 10 (0.0365) and 100 (0.00365) decrease. In the second set, the effect of the same variation in *k*_17_ was tested for a scenario with a five times higher input of organic matter. In the third set, the C_ORG_/P_ORG_ ratio for the refractory γ fraction of OM was varied from 1000 to 4000 mol/mol, with 4000 mol mol^−1^ representing the maximum value seen for sapropel i-282c. In this run, *k*_17_ was set to a value of 0.00365.

### Saturation state modeling

We calculated the porewater SI (which is equal to log Ω_CFA_) for the ancient and modern sediments with the PHREEQC model (*78*) in combination with the Lawrence Livermore National Laboratory (LLNL) database for thermodynamic data, using Davies equations for activity coefficients. Currently, there are no parameters available for phosphate in the PHREEQC Pitzer database. However, for calculations of the SI for other Ca-containing phases such as gypsum, results are comparable when using either the Pitzer or the LLNL database, up to salinities of about 100 (*79*, *80*), because the ion pairs are considered when using the LLNL database, resulting in similar free ion activities. The porewater saturation state was calculated for carbonate-bearing CFA with the following formula

$${\text{Ca}}_{10}{\left({\text{PO}}_{4}\right)}_{5.83–0.57X}{\left({\text{CO}}_{3}\right)}_{X}F_{2.52–0.3X}$$

where *X* is 1.45. This solid composition, with 1.45 carbonate (CO_3_^2−^) for every 10 Ca^2+^, is comparable to a carbonate-rich francolite (*12*, *42*). Solubility coefficients for CFA were taken from (*12*), which includes solubility products (Ksp) for 9°, 14°, and 25°C for PO_4_. Because of the fixed composition of our solid CFA, Ksp only varies with temperature. Ksp can also vary with [CO_3_^2−^]; however, the influence of this is relatively small (*42*).

For this study, Ksp values were recalculated for HPO_4_^2−^ and additional values were extrapolated along an exponential trendline. These values can be used safely between 9° and 25°C. The SIs obtained were calculated by PHREEQC as

SI=log IAP−log Ksp (2)

where IAP is the activity product of the ions making up the CFA. As a result, an SI increase of one unit results in an order of magnitude increase in the degree of supersaturation.

The use of PHREEQC to calculate the saturation state with respect to CFA allows a comparison of geological settings while minimizing the number of assumptions required. For our calculations, we estimate the likely range of values for the pH and [PO_4_], [CO_3_^2−^], and [F^−^] for the chosen paleo-depositional environments, based on what is known about their biogeochemistry. The aim of these PHREEQC calculations is to determine whether porewaters in ancient sediments were likely undersaturated with respect to CFA, effectively preventing the formation of CFA, or whether the degree of supersaturation was insufficient for CFA formation to keep pace with the supply of P to sediments.

### Box model simulation

We illustrate the potential consequences of lower rates of apatite authigenesis for oceanic P and C cycling with results from the ocean box model by (*4*). In our simulations, we used two versions of the model: (i) the original version in which apatite formation is only related to the rate of organic matter decay, following

$${\mathrm{FP}}_{4}={\mathrm{kp}}_{4}\times {{\mathrm{FP}}_{2}}^{2.5}$$ (3)

where FP_4_ is the rate of apatite authigenesis, kp_4_ is a rate constant, and FP_2_ is the rate of organic matter decay [see (*4*) for the full model description]; (ii) a version with a modified rate law that adds a dependency to the formation of authigenic apatite (*1*), which is assumed to reflect a lower rate constant for authigenic apatite formation upon increased anoxia

$${\mathrm{FP}}_{4}=(1-\text{DOA})\times {\mathrm{kp}}_{4}\times {\mathrm{FP}}_{2}$$ (4)

where DOA is the degree of anoxia. A DOA value of 0 describes a fully oxic ocean, whereas a fully anoxic ocean results in a value of 1. Values for C_ORG_/P_ORG_ were varied up to 4000 mol mol^−1^, as was done for the reactive transport modeling.

## Supplementary Materials

### This PDF file includes:

New data Local and global redox conditions Phreeqc sensitivity ranges Sediment CaCO_3_ Fig. S1 Tables S1 to S11 References
