# Supplementary material for: Enhanced phosphorus recycling during past oceanic anoxia amplified by low rates of apatite authigenesis
Source: Sci Adv. 2022 Jul 1;8(26):eabn2370. doi: 10.1126/sciadv.abn2370 (PMC10883373; doi:10.1126/sciadv.abn2370)
Supplement: Supplementary file 1 — New data Local and global redox conditions Phreeqc sensitivity ranges Sediment CaCO3 Fig. S1 Tables S1 to S11 References [file sciadv.abn2370_sm.pdf]

Science Advances
AAAS

# Supplementary Materials for

## Enhanced phosphorus recycling during past oceanic anoxia amplified by low rates of apatite authigenesis

Nina M. Papadomanolaki *et al.*

Corresponding author: Nina M. Papadomanolaki, n.papadomanolaki@uu.nl

*Sci. Adv.* **8**, eabn2370 (2022)
DOI: 10.1126/sciadv.abn2370

**The PDF file includes:**

New data
Local and global redox conditions
Phreeqc sensitivity ranges
Sediment $CaCO_3$
Fig. S1
Tables S1 to S11
Legend for dataset S1
References

**Other Supplementary Material for this manuscript includes the following:**

Dataset S1

## NEW DATA

We include previously unpublished data from earlier work in our data compilation of $C_{ORG}/P_{TOT}$, Fe/Al and $CaCO_3$. The new data for the OAEs are the $CaCO_3$ contents for the T-OAE section at Schandelah (25), the ratios of Fe/Al in OAE2 sediments for Tarfaya S57 and DSDP 530 (81) and (100), the $CaCO_3$ contents for OAE2 sediments for DSDP 641, DSDP 386, ODP 1276 (37) and Bass River (83) and the total P contents for Wunstorf (84). For the sapropels, the relevant data are the $CaCO_3$ contents for KC19C (85) and Fe/Al ratios and $CaCO_3$ contents for MS21PC (86). The methods for the $CaCO_3$, Ca, Fe, Al and P measurements (bulk sediment geochemical analyses) are described in the papers referred to above and involve XRF, dissolution of the sediment followed by ICP-OES or ICP-AES, or decalcification of sediments.

## LOCAL AND GLOBAL REDOX CONDITIONS

Marine deoxygenation is a recurring phenomenon throughout Earth's history, but the lateral and temporal extent of decreasing oxygenations, as well as the degree of deoxygenation, can vary significantly. During the T-OAE, OAE2 and PETM reducing conditions occurred at multiple locations and the extent of severely reducing conditions (anoxia or euxinia) can be estimated from changes in isotopic systems such as molybdenum, uranium and sulphur. Molybdenum isotopic values show that euxinia was less extensive during the PETM, than during the T-OAE and OAE2 (60). During the two OAEs, between 2% and 10% of the ocean seafloor may have been overlain by euxinic bottom waters. The sulfur isotope excursion for the PETM is, at ~1‰ (20), smaller than the 5-7‰ T-OAE excursion (16) and 2-6‰ OAE2 excursion (17) and,

therefore, also reflects less extensive euxinia during the former event. Specifically, for the PETM the sulfur isotope excursion is interpreted as an increase in the volume of Oxygen Minimum Zones ($O_2 < 20$ μM) by a factor 10 to 20. For the two OAEs, the respective excursions indicate a seafloor area extent of euxinia similar to the range predicted by molybdenum isotopes. Uranium isotope records also allow for a comparison between OAE2 and the PETM, in terms of seafloor anoxia extent. For the PETM, uranium isotopes constrain the maximum extent of anoxia at 2% of the seafloor (61). While (87) estimate a similar extent for OAE2 from their uranium isotope record, (59) use new uranium isotope records to model a maximum seafloor anoxia extent of 15% for the same event. The deposition of sapropels was restricted mostly to the eastern Mediterranean and their extent can therefore not be compared easily to large-scale events such as the OAEs and the PETM. Redox conditions can, however, be compared between the different sapropels. Specifically, uranium and molybdenum isotope values for the S1 and S5 sapropels indicate that conditions during S5 were more strongly reducing than during S1 (36). During peak sapropel conditions, anoxic seafloor conditions during S5 occurred above 1000m whereas for S1 the remained well below this (31). Specifically for the S5, the chemocline likely occurred around 150-300m with proxy records indicating euxinia in the photic zone (31). For the S1, a well-document oxygenation event splits the sapropel layer in two parts (S1a and S1b) (31 and references therein).

The redox conditions for the sites discussed in this study (chosen for their availability of $C_{ORG}/P_{TOT}$ and other redox data) vary (see **Table S11**, also for references). For the T-OAE, proxy records indicate at least intermittent euxinia at all four sites. Even intermittent euxinia, taking into consideration the long duration of the two events, could mean persistence of euxinia over thousands to tens of thousands of years. During OAE2, there is evidence of photic zone euxinia for the majority of our sites. All sites, with the exception of ODP Site 1276, Wunstorf and Bass River, experienced water column euxinia and only Wunstorf and Bass River were likely sulfide free even in the sediments. At Tarfaya and Wunstorf, the deposition of black shales and the associated redox conditions varied on orbital timescales (tens of thousands of years). Molybdenum values at four sites, DSDP Sites 367 and 530, and ODP Sites 641 and 1260, indicate permanent euxinia. We note that for the T-OAE and OAE2 molybdenum values are likely affected by

basinal restriction (37,72). As a result, even when Mo values are below the 100 ppm threshold, it's possible that they did in fact experience persistently euxinic conditions. In contrast to the T-OAE and OAE2, only three PETM sites show indications of being intermittently to permanently euxinic (the Arctic, Kheu River and Guru Fatima), while photic zone euxinia occurred (intermittently or for longer periods of time) at two sites (the Arctic and IB10). All other sites had at most sulfidic porewaters. At our single i-282c site conditions were permanently euxinic with photic zone euxinia occurring as well. One of the S5 sites (PS25PC) was intermittently to permanently euxinic, while KC19C was likely at least anoxic. For S1, KC19C may have been anoxic and at MS21PC porewaters, and potentially bottom waters, were sulfidic.

## PHREEQC SENSITIVITY RANGES

We used measured porewater concentrations for the modern sediments and estimated values for the ancient sediments as input for the PHREEQC calculations, as detailed below. Obviously, porewater and seawater concentrations and environmental characteristics for ancient marine systems cannot be measured directly and hence must be derived from, e.g., proxy and modelling studies. Here, we use a range of values for [$PO_4$], pH, temperature and key solute concentrations. Where possible, these ranges are based on estimates for seawater and porewater values from earlier studies.

For the modern Arabian Sea and Black Sea, porewater concentrations were obtained through sampling and analyses in 2009 (32) and 2015 (11), respectively. For OAE2, the PETM, and the i-282c and S5 sapropels, we estimated minimum and maximum porewater concentrations of major ions from model and proxy data for bottom waters (**Table S4**). Note that we did not include the T-OAE because of a lack of sufficient data on seawater composition. For [$PO_4$], our range (1 μmol $l^{-1}$ and 11 μmol $l^{-1}$) captures the values used by (88) for OAE2 simulations, the low bottom water [$PO_4$] of the modern SE Mediterranean (~2 μmol $l^{-1}$) (51), the maximum porewater concentrations estimated for sapropel S1 based on diagenetic modelling (6.5 μmol $l^{-1}$) (89) and the average value for modern porewaters in sediments of the euxinic basin of the Black Sea (~10 μmol $l^{-1}$), where CFA authigenesis occurs. For OAE2, we also tested the effect of the much higher [$PO_4$] concentrations that are found in porewaters of the Arabian Sea (80 μmol $l^{-1}$). Our range

of values is thus wide enough to account for both low [$PO_4$] in porewaters in deep sea sediments and increased porewater [$PO_4$] following deoxygenation.

The maximum pH values used for OAE2 (7.6) and the PETM (7.7) correspond to the estimated, average ocean pH during their respective time periods (23), while for sapropels the value corresponds to the water column pH of the modern Eastern Mediterranean (8.2) (55). The minimum pH value used for all ancient sediments is 6.9 and accounts for the generally lower pH values in porewaters (56). These ranges cover the pH values for modern marine surface sediments (top 10 cm) (23).

The chosen temperature range for OAE2 is based on proxy and model reconstructions of temperature during the event (48,49,90,91). The range of bottom water temperatures for the PETM is similar to OAE2 (92,93). Therefore we use a minimum temperature of 14˚C and a maximum temperature of 25˚C for both events. Sapropel i-282c was deposited during a period of warming (54) and the deposition of sapropel S5 corresponds to a solar insolation maximum (94). We, therefore, assign a maximum temperature of 17˚C to our sapropel calculations, which is a few degrees higher than the modern bottom value of 14˚C for the Eastern Mediterranean south of Crete (55). The full list of values and their references can be found in **Table S4**.

We also tested the effect of changes in alkalinity and [$Ca^{2+}$], [$Mg^{2+}$] and [$SO_4^{2-}$] on the saturation index of CFA using the corresponding average ocean value for each time period (23). Overall the effects of changes in these parameters were small to negligible when compared to those of temperature, pH and [$PO_4$]. For OAE2, an increase in [$Ca^{2+}$] from 17 mmol Ca $kg^{-1}$ to 29 mmol Ca $kg^{-1}$, the maximum range tested here, had the largest effect of the three ion concentrations, yet we only observed an increase in SI of ~2 units. Furthermore, as ocean [$Ca^{2+}$] during the ancient events (>10 mmol Ca $l^{-1}$) was higher than that in the modern Arabian Sea (~10 mmol Ca $l^{-1}$) and Black Sea (~7 mmol Ca $l^{-1}$), changes in [$Ca^{2+}$] cannot have contributed to the reduction in CFA authigenesis during past deoxygenation events.

Within the ranges tested, the effect of temperature on SI was an increase of ~13 units for OAE2 and the PETM and ~3 units for the sapropels, or an increase of roughly 1 unit per 1˚C. Similarly, the effect of a change in pH was 4.7 for OAE2, 5.3 for the PETM and ~8 for the sapropels. Within the 1 – 11 μmol $l^{-1}$

range for $[PO_4]$, the change in SI was equal to 5 for all ancient sediments. However, as we note in the main text, the effect of changes in $[PO_4]$ was largest at low concentrations, whereas the effect of a change in pH and temperature remained constant. This is why, even with much higher $[PO_4]$ (80 μmol $l^{-1}$), the change in SI is only 4 units. For the sapropels, due to the larger range for pH and the smaller temperature range, the effect of $[PO_4]$ is relatively more important. However, for OAE2 and the PETM the combined effect of temperature and pH is more significant, producing lower SI values irrespective of the $[PO_4]$.

## SEDIMENT $CaCO_3$

Average $CaCO_3$ contents for all sites are given in **Table S5**. Profiles of $C_{ORG}/P_{TOT}$ and $CaCO_3$ for sapropel i-282c (ODP Site 969) (38) are shown in **Fig. S1**.

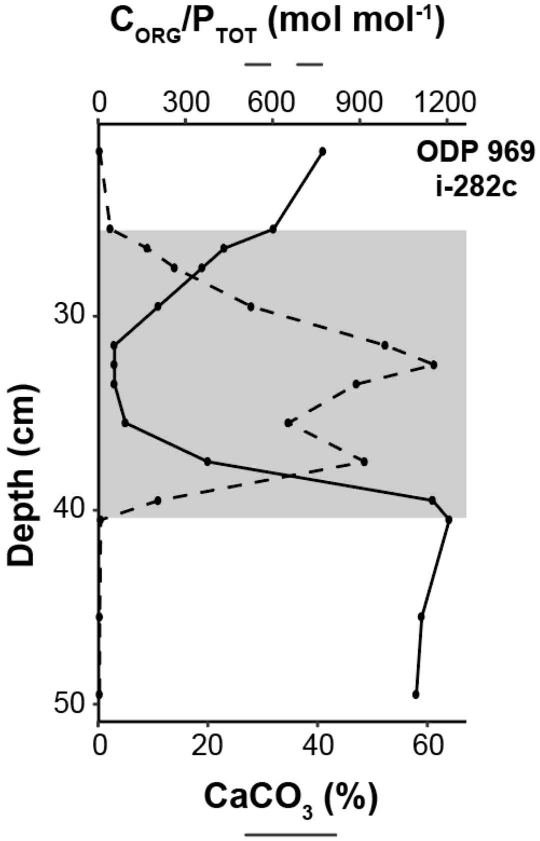

**Supplementary Figure S1.** Calcium carbonate content ($CaCO_3$; solid line) and $C_{ORG}/P_{TOT}$ values (dashed line) for sapropel i-282c sediments from ODP Site 969 (38). The grey area indicates the position of the sapropel.

**Table S1.** Complete list of sources for the data shown in: Fig. 1 ($C_{org}/P_{tot}$), Fig. 2 ($C_{org}/P_{tot}$, Fe/Al and Mo) and Supp. Fig. 3 ($CaCO_3$). Reference numbers correspond to the reference list in the main text. Note that for previously unpublished data (indicated as *This study*) we provide the reference number to the original study for which these were measured between double brackets.

| Site | Event/Basin | Depositional setting | $C_{org}/P_{tot}$ (mol/mol) | Fe/Al (%/%) | Mo (ppm) | $CaCO_3$ (%) |
|---|---|---|---|---|---|---|
| Schandelah | T-OAE | Epicontinental shelf | (25) | (25) | (25) | This study ((25)) |
| Dotternhausen | | | (95) ((25)) | (95) ((25)) | (25) | (95) ((25)) |
| Rietheim | | | (96) | (96) ((25)) | | (96) |
| Yorkshire | | | (95) ((25)) | (95) ((25)) | (25) | (95) ((25)) |
| ODP 1260 | OAE2 | Continental slope | (9) | (97) | (97) | (97) |
| Tarfaya S57 | | Continental shelf | (9) | This study ((81)) | (9) ((81)) | This study ((81)) |
| DSDP 641 | | Deep ocean | (37) | (37) | (37) | This study |
| DSDP 603 | | Deep ocean | (9) | (98) | (9) ((82)) | (9) |
| DSDP 386 | | Deep ocean | (37) | (37) | (37) | This study |
| DSDP 367 | | Deep ocean | This study ((37)) | (98) | (99) | (40) |
| ODP 1276 | | Deep ocean | (37) | (37) | (37) | This study |
| DSDP 530 | | Deep ocean | (9) | This study ((100)) | (100) | (100) |
| Bass River | | Continental shelf | (83) | (83) | This study ((83)) | This study ((83)) |
| Wunstorf | | Epicontinental shelf | This study ((84)) | (84) | (101) | (101) |
| Bass River | PETM | Continental shelf | (57) | (57) | (57) | (102) |

| | | | | | | |
|---|---|---|---|---|---|---|
| IODP M006, ACEX | | Continental shelf | (57) | (103) | (57) | (103) |
| IB10 | | Epicontinental shelf | (104) | (104) | (104) | (104) |
| ODP 959 | | Continental slope | (105) | (105) | (105) | (105) |
| IODP 1403 | | Deep ocean | (57) | (57) | (57) | (106) |
| ODP 1172 | | Continental shelf | (57) | (57) | (57) | (107) |
| ODP 752 | | Continental slope | (57) | (57) | (57) | (108) |
| Forada | | Continental slope | (109) | (57) | (57) | (109) |
| Dzhengutay | | Epicontinental shelf | (28) | (28) | (28) | |
| Guru Fatima | | Epicontinental shelf | (28) | (28) | (28) | |
| Kheu River | | Epicontinental shelf | (28) | (28) | (28) | |
| 969 | i-282c | Deep ocean | (38) | (38) | This study ((38)) | (38) |
| PS25PC | S5 | Deep ocean | (39) | This study | (39) | This study |
| KC19C | | Deep ocean | (39) | (39) (85) | | This study |
| MS21PC | S1 | Deep ocean | (86) | This study (86) | (86) | This study (86) |
| KC19C | | Deep ocean | (39) | (39) (85) | | This study |
| 1B (OMZ)<br>2 (OMZ)<br>3<br>4<br>5<br>6B | Arabian Sea | Continental slope (Oxygen Minimum Zone) | (32) | | | |

| | | | |
|---|---|---|---|
| 7 | | | |
| 8 | | | |
| 9 | | | |
| 10 | | | |
| 2 (Anoxic) | Black Sea | Deep ocean | (11) |
| 3 (Anoxic) | | | |
| 4 (Anoxic) | | Continental slope | |
| 6A | | | |
| 6B | | | |
| 7 | | Continental shelf | |

**Table S2**. Median and maximum $C_{org}/P_{tot}$ values for the Black Sea, Arabian Sea, T-OAE, OAE2, PETM, and i-282c, S5 and S1 sapropels as shown in Fig. 1. For references, see Table S1.

| **Area/Event** | | **$C_{org}$:$P_{tot}$ (mol/mol)** | |
|---|---|---|---|
| | | **Median** | **Maximum** |
| T-OAE | pre- | 44 | 327 |
| | event | 169 | 1099 |
| OAE2 | pre- | 28 | 554 |
| | event | 100 | 1835 |
| PETM | pre- | 16 | 118 |
| | event | 47 | 299 |
| i-282c | oxic | 6 | 9 |
| | sapropel | 655 | 1156 |
| S5 | oxic | 18 | 55 |
| | sapropel | 315 | 698 |
| S1 | oxic | 27 | 50 |
| | sapropel | 53 | 187 |
| Arabian Sea | below OMZ | 38 | 97 |
| | OMZ | 103 | 151 |
| Black Sea | oxic | 64 | 130 |
| | euxinic | 189 | 354 |

**Table S3.** Median $C_{org}/P_{tot}$ (mol/mol), Fe/Al (%/%) and Mo (ppm) values for the T-OAE, OAE2, PETM, and i-282c, S5 and S1 sapropels as shown in Fig. 2. For references, see Table S1. Numbers correspond to those given on the vertical axis of Fig. 2.

| Event | Site | Number | $C_{org}/P_{tot}$ | Fe/Al | Mo |
|---|---|---|---|---|---|
| TOAE | Schandelah | 8 | 272 | 0.81 | 14.3 |
| | Dotternhausen | 10 | 194 | 0.71 | 18.5 |
| | Rietheim | 12 | 157 | 0.74 | - |
| | Yorkshire | 17 | 109 | 0.60 | 9 |
| OAE2 | ODP 1260 | 7 | 331 | 0.80 | 32 |
| | Tarfaya S57 | 3 | 507 | 0.63 | 9.5 |
| | DSDP 641 | 14 | 142 | 0.55 | 2139 |
| | DSDP 603 | 9 | 248 | 0.62 | 15 |
| | DSDP 386 | 1 | 844 | 1.44 | 27.3 |
| | DSDP 367 | 5 | 405 | 1.00 | 89 |
| | ODP 1276 | 13 | 144 | 0.67 | 3 |
| | DSDP 530 | 16 | 122 | 1.06 | 16 |
| | Bass River | 19 | 100 | 0.43 | 0.7 |
| | Wunstorf | 28 | 7 | 0.58 | 0 |
| PETM | Bass River | 25 | 40 | 0.22 | 0.3 |
| | IODP M006, ACEX | 15 | 131 | 0.52 | 32 |
| | IB10 | 26 | 23 | 0.39 | 2.1 |
| | ODP 959 | 23 | 56 | 0.47 | - |
| | IODP 1403 | 30 | 5 | 0.75 | 0 |
| | ODP 1172 | 24 | 40 | 1.00 | 1.2 |
| | ODP 752 | 27 | 11 | 0.60 | 0 |
| | Forada | 29 | 7 | 0.57 | 0 |
| | Dzhengutay | 22 | 59 | 0.45 | 59 |
| | Guru Fatima | 18 | 106 | 0.44 | 105.8 |
| | Kheu River | 20 | 85 | 0.61 | 84.9 |

| | | | | | |
|---|---|---|---|---|---|
| i-282c | 969 | 2 | 655 | 1.26 | 453.5 |
| S5 | PS25PC | 6 | 340 | 1.12 | 114.4 |
| | KC19C | 4 | 438 | 1.02 | - |
| S1 | MS21PC | 21 | 61 | 0.71 | 14 |
| | KC19C | 11 | 161 | 1.16 | - |

**Table S4**. Chemical species included in the reactive transport model.

| SPECIES | NOTATION |
|---|---|
| **Solid** | |
| Organic Matter[a] | $OM^{\alpha,\beta,\gamma}$ |
| Iron oxides[a] | $Fe(OH)_3^{\alpha,\beta,\gamma}$ |
| Manganese oxides[a] | $Mn(OH)_2^{\alpha,\beta,\gamma}$ |
| Elemental Sulfur | $S^0$ |
| Iron monosulfide | FeS |
| Pyrite | $FeS_2$ |
| Authigenic calcium phosphates | Auth. Ca-P |
| **Solute** | |
| Oxygen | $O_2$ |
| Nitrate | $NO_3^-$ |
| Sulfate | $SO_4^{2-}$ |
| Methane | $CH_4$ |
| Iron | $Fe^{2+}$ |
| Manganese | $Mn^{2+}$ |
| Ammonium[b] | $\Sigma NH_4^+$ |
| Phosphate[c] | $\Sigma PO_4$ |
| Hydrogen sulfide[d] | $\Sigma H_2S$ |

[a]Chemical species consist of three types: reactive (α), less reactive (β) and refractory (γ).

[b]$\Sigma NH_4^+ = NH_4^+ + NH_3$

[c]$\Sigma PO_4 = H_3PO_4 + H_2PO_4^- + HPO_4^{2-} + PO_4$

[d]$\Sigma H_2S = H_2S + HS^- + S^{2-}$

**Table S5.** Reaction pathways and stoichiometries implemented in the model.

| REACTION FORMULA | CODE |
|---|---|
| **Primary redox reactions** | |
| $OM^{\alpha,\beta} + aO_2 \rightarrow aCO_2 + bNH_4^+ + cPO_4 + H_2O$ | R1 |
| $OM^{\alpha,\beta} + 0.8aNO_3^- + 0.8H^+ \rightarrow aCO_2 + bNH_4^+ + cPO_4 + 0.4aN_2 + 1.4aH_2O$ | R2 |
| $OM^{\alpha,\beta} + 2aMn(OH)_2^{\alpha} + 2aH^+ \rightarrow 2Mn^{2+} + aCO_2 + bNH_4^+ + cPO_4 + 2aH_2O$ | R3 |
| $OM^{\alpha,\beta} + 4aFe(OH)_3^{\alpha} + 4a\chi Fe\text{-}P^{\alpha} + 12aH^+ \rightarrow aCO_2 + bNH_4^+ + (c+4a\chi)PO_4 + 13aH_2O + 4aFe^{2+}$ | R4 |
| $OM^{\alpha,\beta} + 0.5aSO_4^{2-} + aH^+ \rightarrow aCO_2 + bNH_4^+ + cPO_4 + 0.5aH_2S + aH_2O$ | R5 |
| $OM^{\alpha,\beta} \rightarrow 0.5aCO_2 + bNH_4^+ + cPO_4 + 0.5CH_4$ | R6 |
| **Secondary and other reactions** | |
| $2O_2 + NH_4^+ + 2HCO_3^- \rightarrow NO_3^- + 2CO_2 + 3H_2O$ | R7 |
| $O_2 + 2Mn^{2+} \rightarrow 2aMn(OH)_2^{\alpha} + 4CO_2 + 2H_2O$ | R8 |
| $O_2 + 4Fe^{2+} + 8HCO_3^- + 2H_2O + 4\chi PO_4 \rightarrow 4aFe(OH)_3^{\alpha} + 4a\chi Fe\text{-}P^{\alpha} + 8CO_2$ | R9 |
| $2O_2 + FeS \rightarrow SO_4^{2-} + Fe^{2+}$ | R10 |
| $7O_2 + 2FeS_2 + 2H_2O \rightarrow 4SO_4^{2-} + 2Fe^{2+} + 2H_2O$ | R11 |
| $2O_2 + H_2S + 2HCO_3^- \rightarrow SO_4^{2-} + 2CO_2 + 2H_2O$ | R12 |
| $O_2 + CH_4 \rightarrow CO_2 + 2H_2O$ | R13 |
| $Mn(OH)_2^{\alpha,\beta} + 2Fe^{2+} + 2\chi PO_4 + 2H_2O + 2HCO_3^- \rightarrow 2aFe(OH)_3^{\alpha} + 2\chi Fe\text{-}P^{\alpha} + S^0 + 4HCO_3^- + 2H_2O$ | R14 |
| $Mn(OH)_2^{\alpha,\beta} + H_2S + 2CO_2 \rightarrow Mn^{2+} + S^0 + 2HCO_3^-$ | R15 |

| | |
|---|---|
| $2Fe(OH)_3^{\alpha,\beta} + 2\chi Fe\text{-}P^{\alpha,\beta} + H_2S + 4CO_2 \rightarrow 2Fe^{2+} + 2\chi PO_4 + S^0 + 4HCO_3^- + 2H_2O$ | R16 |
| $Fe^{2+} + H_2S \rightarrow FeS + 2H^+$ | R17 |
| $SO_4^{2-} + CH_4 + CO_2 \rightarrow 2HCO_3^{2-} + H_2S$ | R18 |
| $4S^0 + 4H_2O \rightarrow 3H_2S + SO_4^{2-} + 2H^+$ | R19 |
| $FeS + S^0 \rightarrow FeS_2$ | R20 |
| $Fe(OH)_3^{\alpha} + \chi Fe\text{-}P^{\alpha} \rightarrow Fe(OH)_3^{\beta} + \chi Fe\text{-}P^{\beta}$ | R21 |
| $Mn(OH)_2^{\alpha} \rightarrow Mn(OH)_2^{\beta}$ | R22 |
| $PO_4 \rightarrow Ca\text{-}P$ | R23 |
| $OM^{\beta} \rightarrow OM^{\gamma} + (OM^{\gamma} * 1/CtoP^{\beta} - OM^{\gamma} * 1/CtoP^{\gamma}) * PO_4$ | R24 |

**Tabel S6.** Reaction equations implemented in the reactive transport model.

| EQUATION | CODE |
|---|---|
| **Primary redox reaction equations** | |
| $R1 = K_{\alpha,\beta}\, OM^{\alpha,\beta}$ | E1 |
| $R2 = K_{\alpha,\beta}\, OM^{\alpha,\beta}$ | E2 |
| $R3 = K_{\alpha,\beta}\, OM^{\alpha,\beta}$ | E3 |
| $R4 = K_{\alpha,\beta}\, OM^{\alpha,\beta}$ | E4 |
| $R5 = K_{\alpha,\beta}\, OM^{\alpha,\beta}$ | E5 |
| $R6 = K_{\alpha,\beta}\, OM^{\alpha,\beta}$ | E6 |
| **Secondary redox and other reaction equations** | |
| $R6= k_1[O_2][\sum NH_4^+]$ | E7 |
| $R8= k_2[O_2][Mn^{2+}]$ | E8 |
| $R9= k_3[O_2][Fe^{2+}]$ | E9 |
| $R10= k_4[O_2][FeS]$ | E10 |
| $R11= k_5[O_2][FeS_2]$ | E11 |
| $R12= k_6[O_2][H_2S]$ | E12 |
| $R13= k_7[O_2][CH_4]$ | E13 |
| $R14= k_8[Mn(OH)_2^{\alpha,\beta}][Fe^{2+}]$ | E14 |
| $R15= k_9[Mn(OH)_2^{\alpha,\beta}][\sum H_2S]$ | E15 |
| $R16 = k_{10}[Fe(OH)_3^{\alpha,\beta}][H_2S]$ | E16 |

| | |
|---|---|
| $R17 = k_{11}[Fe^{2+}][H_2S]$ | E17 |
| $R18 = k_{12}[SO_4^{2-}][CH_4]$ | E18 |
| $R19 = k_{13}[S_0]$ | E19 |
| $R20 = k_{14}[FeS][S_0]$ | E20 |
| $R21 = k_{15}[Fe(OH)_3^{\alpha}]$ | E21 |
| $R22 = k_{16}[Mn(OH)_2^{\alpha}]$ | E22 |
| $R23 = k_{17}[\sum PO_4]$ | E23 |
| $R24 = k_{18}[OM^{\beta}]$ | E24 |

**Table S7.** Key environmental parameters, elemental ratios and boundary conditions used in the reactive transport model relevant to the scenario. All other settings were as in (32).

| DESCRIPTION | SYMBOL | VALUE/EXPRESSION | UNIT | SOURCE |
|---|---|---|---|---|
| Porosity at the surface | $\Phi_0$ | 0.86 | vol vol $^{-1}$ | a |
| Porosity at depth | $\Phi_\infty$ | 0.76 | vol vol $^{-1}$ | a |
| Porosity e-folding distance | $\gamma$ | 6.63 | cm | a |
| Sediment density | $\rho$ | 2.65 | g $cm^{-3}$ | a |
| Temperature | T | 6.7 | °C | a |
| Salinity | S | 36.1 | | a |
| Pressure | P | 131.1 | Bar | a |
| Advective velocity of solids at depth | $v_\infty$ | | | a |
| P:Fe ratio for iron-bound phosphate | X | 0.16 | mol $mol^{-1}$ | a |
| C:N ratio for $OM^{\alpha}$ | $CtoN^{\alpha}$ | 6.25 | mol $mol^{-1}$ | a |
| C:N ratio for $OM^{\beta,\gamma}$ | $CtoN^{\beta,\gamma}$ | 9.54 | mol $mol^{-1}$ | a |
| C:P ratio for $OM^{\alpha,\beta}$ | $CtoP^{\alpha,\beta}$ | 106 | mol $mol^{-1}$ | b |
| C:P ratio for $OM^{\gamma}$ | $CtoP^{\gamma}$ | 1200 | mol $mol^{-1}$ | c |
| Sedimentation rate | | 13.4 | cm $kyr^{-1}$ | d |
| Bioirrigation constant | | 0 | $yr^{-1}$ | e |
| Bioturbation coefficient | | 0 | $cm^2$ $yr^{-1}$ | e |

| | | | | |
|---|---|---|---|---|
| Bottom water oxygen | | 0 | µmol $L^{-1}$ | e |
| $OM^{\alpha}$ flux to SWI | | 12.6 | µmol $cm^{-2}$ $yr^{-1}$ | d |
| $OM^{\beta}$ flux to SWI | | 14.9 | µmol $cm^{-2}$ $yr^{-1}$ | d |
| $Fe(OH)_3^{\alpha}$ flux to SWI | | 0.13 | µmol $cm^{-2}$ $yr^{-1}$ | d |
| $Mn(OH)_2^{\alpha}$ flux to SWI | | 1.75 | µmol $cm^{-2}$ $yr^{-1}$ | d |

a: (32); b: (35); c: based on range for modern sediments, Fig. 3; d: based on sedimentation rate for site 1B in OMZ in (32); e: no activity of benthic fauna and no oxygen in fully anoxic setting.

**Table S8.** Reaction parameters used in the model.

| PARAMETER | SYMBOL | VALUE | UNIT | SOURCE |
|---|---|---|---|---|
| Decay constant $OM^{\alpha}$ | $k_{\alpha}$ | 0.25 | $yr^{-1}$ | a |
| Decay constant $OM^{\beta}$ | $k_{\beta}$ | 0.0015 | $yr^{-1}$ | a |
| Limiting concentration of $O_2$ | | 20 | µmol $L^{-1}$ | a |
| Limiting concentration of $NO_3^-$ | | 4 | µmol $L^{-1}$ | a |
| Limiting concentration of $Mn(OH)_2$ | | 4 | µmol $g^{-1}$ | a |
| Limiting concentration of $Fe(OH)_3$ | | 65 | µmol $g^{-1}$ | a |
| Limiting concentration of $SO_4^{2-}$ | | 1.6 | mmol $L^{-1}$ | a |
| Attenuation factor for $SO_4^{2-}$ reduction | | 0.075 | | a |
| Rate constant E7 | $k_1$ | 500.000 | mmol $L^{-1}$ $yr^{-1}$ | a |
| Rate constant E8 | $k_2$ | 20.000 | mmol $L^{-1}$ $yr^{-1}$ | a |
| Rate constant E9 | $k_3$ | 140.000 | mmol $L^{-1}$ $yr^{-1}$ | a |
| Rate constant E10 | $k_4$ | 300 | mmol $L^{-1}$ $yr^{-1}$ | a |
| Rate constant E11 | $k_5$ | 1 | mmol $L^{-1}$ $yr^{-1}$ | a |
| Rate constant E12 | $k_6$ | 160 | mmol $L^{-1}$ $yr^{-1}$ | a |
| Rate constant E13 | $k_7$ | 10.000.000 | mmol $L^{-1}$ $yr^{-1}$ | a |
| Rate constant E14 | $k_8$ | 2 | mmol $L^{-1}$ $yr^{-1}$ | a |
| Rate constant E15 | $k_9$ | 20 | mmol $L^{-1}$ $yr^{-1}$ | a |
| Rate constant E16 | $k_{10}$ | 0.15 | mmol $L^{-1}$ $yr^{-1}$ | a |

| | | | | |
|---|---|---|---|---|
| Rate constant E17 | $k_{11}$ | 0.5 | mmol $L^{-1}$ $yr^{-1}$ | a |
| Rate constant E18 | $k_{12}$ | 10 | mmol $L^{-1}$ $yr^{-1}$ | a |
| Rate constant E19 | $k_{13}$ | 3 | $yr^{-1}$ | a |
| Rate constant E20 | $k_{14}$ | 7 | mmol $L^{-1}$ $yr^{-1}$ | a |
| Rate constant E21 | $k_{15}$ | 0.6 | $yr^{-1}$ | a |
| Rate constant E22 | $k_{16}$ | 1.8 | $yr^{-1}$ | a |
| Rate constant E23 | $k_{17}$ | 0.365 | $yr^{-1}$ | a |
| Rate constant E24 | $k_{18}$ | 0.01 | $yr^{-1}$ | b |

a: (32); b: Model constrained

**Table S9.** Input values for the PHREEQC Saturation Index (SI) calculations as shown in Fig. 3A. For OAE2, the PETM and the sapropels, a number of different combinations were tested, with values and SI results that fall between the ones presented in this table. For the Black Sea and Arabian Sea, calculations were performed for four depths between 0.25 cm and 32.5 cm. The average SI value for each location is shown here. Numbers between brackets refer to references as given below the table. Full references are given in the Supplementary Information.

| **Basin/ Event** | **Site** | **Description** | **T (˚C)** | **pH** | **$PO_4$ (µmol/ l)** | **Ca (mmol/ kg)** | **Alk (mmol/ kg)** | **Mg (mmol/ kg)** | **$SO_4$ (mmol/ kg )** | **SI** |
|---|---|---|---|---|---|---|---|---|---|---|
| OAE2 | | Min[a] | 25[1] | 6.9[2,*] | 1.1[3] | 17[2] | 2[2] | 40[2] | 14[2] | -1.4 |
| | | Max[a] | 14[1] | 7.6[2] | 11[3] | 29[2] | 2.5[2] | 30[2] | 8[2] | 24.1 |
| | | Test | | | 80 | | | | | 28.6 |
| PETM | | Min[a] | 25[4,5] | 6.9[6,*] | 2.5[6] | 15[2] | 2[6] | 40[2] | 15[2] | -1.94 |
| | | Max[a] | 14[4,5] | 7.7[6] | 3[6] | 22[2] | 2.5[6] | 30[2] | 10[2] | 23.6 |
| Sapropels | | Min[a] | 17[7-11] | 6.9[*] | 1[7] | 10[2] | 2[2] | 50[2] | 26[2] | 6.3 |
| | | Max[a] | 14[7-11] | 8.2[12,13] | 6.5[14] | 13[2] | 3[2] | 45[2] | 23[2] | 23.9 |
| Arabian Sea | Station 1B | Modern[b] | 10[17] | 7.65[17] | 66.7[17] | ~10[17] | 3.3[17] | 77.8[17] | 28.5[17] | 26.8 |
| | | Future | 25 | 6.9 | | | | | | 4.3 |
| Black Sea | Station 2 | Modern[b] | 9.1[15] | 7.43[16] | 10.3[15] | 6.64[15] | 5[15] | 32.4[15] | 16.3[15] | 24.2 |
| | | Future | 25 | 6.9 | | | | | | 3.4 |

[a]Values that result in minimum and maximum SI values, [b]Depth averaged values, [*](56), [1](49), [2](23), [3](88), [4](92), [5](93), [6](109), [7](55), [8](54), [9](28), [10](110), [11](53), [12](111), [13](112), [14](89), [15](11), [16]Average value for top 5 cm of Black Sea st. 2 in (113), [17](32)

**Table S10.** Calcium carbonate contents ($CaCO_3$: average, maximum, minimum) for all sites presented in Fig. 2. For the T-OAE, OAE2, PETM and for sapropels, the pre-event/sapropel values are also given. The values presented for the Arabian Sea and Black Sea cover the sampled depths at each station. References are listed in Table S1.

| Site | Event/Basin | $CaCO_3$ | | | | | |
|---|---|---|---|---|---|---|---|
| | | Pre-event/sapropel | | | Event or sapropel | | |
| | | Average | Maximum | Minimum | Average | Maximum | Minimum |
| Schandelah | T-OAE | 0 | 0 | 0 | 20 | 41 | 0 |
| Dotternhausen | | 26 | 42 | 12 | 33 | 79 | 11 |
| Rietheim | | 40 | 55 | 19 | 23 | 47 | 6 |
| Yorkshire | | 3 | 8 | 0.5 | 8 | 35 | 1 |
| ODP 1260 (NIOZ) | OAE2 | 69 | 98 | 55 | 60 | 94 | 23 |
| ODP 1260 | | 62 | 96 | 49 | 53 | 92 | 5 |
| Tarfaya S57 (Bremen) | | 76 | 100 | 50 | 84 | 100 | 28 |
| Tarfaya S57 (ICBM) | | 65 | 92 | 40 | 74 | 92 | 50 |
| DSPD 641 | | 26 | 39 | 16 | 18 | 30 | 12 |
| DSDP 603 | | 0.9 | 2 | 0.3 | 0.5 | 2 | 0.2 |
| DSDP 386 | | 7 | 30 | 0.9 | 10 | 14 | 7 |
| DSDP 367 | | 5 | 22 | 1 | 8 | 20 | 0.6 |
| ODP 1276 | | 17 | 33 | 0.8 | 1 | 9 | 0 |
| DSDP 530 | | 1 | 18 | 0 | 0.6 | 9 | 0 |
| Bass River | | 14 | 18 | 12 | 17 | 26 | 10 |
| Wunstorf | | 10 | 11 | 10 | 8 | 11 | 2 |
| Bass River | PETM | 5 | 8 | 1 | 10 | 21 | 0.95 |
| IODP M006, ACEX | | 0.3 | 0.4 | 0.2 | 0.3 | 0.5 | 0.1 |
| IB10 | | 9 | 42 | 1 | 3 | 7 | 2 |

| | | | | | | | |
|---|---|---|---|---|---|---|---|
| ODP 959 | | 13 | 25 | 3 | 0 | 0 | 0 |
| ODP 1403 | | 0.4 | 0.7 | 0.3 | 17 | 33 | 0.2 |
| ODP 1172 | | 1.11 | 9.50 | 0.30 | 0.94 | 1.61 | 0.41 |
| ODP 752 | | | | | 3 | 11 | 0 |
| Forada | | 47 | 60 | 28 | 36 | 74 | 0 |
| 969 | i-282c | 61 | 65 | 58 | 16 | 61 | 3 |
| PS25PC | S5 | 38 | 41 | 34 | 35 | 42 | 26 |
| KC19C | | 50 | 52 | 47 | 39 | 44 | 29 |
| MS21PC | S1 | 12 | 15 | 9 | 14 | 29 | 9 |
| KC19C | | 41 | 52 | 38 | 38 | 40 | 36 |

| **Site** | **Event/Basin** | **$CaCO_3$** | | | |
|---|---|---|---|---|---|
| | | **Pre-event/sapropel** | **Event or sapropel** | | |
| | | - | **Average** | **Maximum** | **Minimum** |
| 1B (Carbonates) | Arabian Sea | - | 53 | 59 | 44 |
| 2 | | | 59 | 61 | 57 |
| 3 | | | 62 | 64 | 60 |
| 4 | | | 58 | 60 | 57 |
| 5 | | | 71 | 73 | 69 |
| 6B | | | 63 | 66 | 59 |
| 7 | | | 46 | 51 | 44 |
| 8 | | | 47 | 50 | 45 |
| 9 | | | 42 | 47 | 40 |
| 10 | | | 43 | 48 | 40 |
| 1B (from Ca) | | | 27 | 33 | 21 |

| | | | | | |
|---|---|---|---|---|---|
| 2 | | | 45 | 51 | 41 |
| 3 | | | 49 | 51 | 47 |
| 4 | | | 45 | 50 | 40 |
| 5 | | | 62 | 66 | 56 |
| 6B | | | 52 | 56 | 45 |
| 7 | | | 30 | 35 | 26 |
| 8 | | | 47 | 50 | 45 |
| 9 | | | 26 | 33 | 21 |
| 10 | | | 27 | 36 | 21 |
| 2 | Black Sea | - | 52 | 81 | 18 |
| 3 | | | 42 | 69 | 11 |
| 4 | | | 64 | 64 | 9 |
| 6A | | | 50 | 88 | 17 |
| 6B | | | 45 | 94 | 13 |
| 7 | | | 63 | 86 | 30 |

**Table S11.** Compilation of available redox condition indicators for the sites used in this study. The indicators presented here are used specifically to distinguish between oxic, hypoxic, anoxic and euxinic conditions, using the interpretations from the studies in which each indicator was published. The redox interpretation for molybdenum concentrations is based on (34). Note that molybdenum concentration values for the T-OAE and OAE2 may be affected by basinal restriction.

| SITE | EVENT | PZE* | DEGREE OF PYRITIZATION | | ISOTOPES | | MOLYBDENUM | | | OXYGENATION INDICATORS | | OTHER INDICATORS | |
|---|---|---|---|---|---|---|---|---|---|---|---|---|---|
| | | | Redox state | Study. | Redox state | Study. | Concen-tration | Redox state | Study. | Proxy | Study. | Proxy/Redox State | Study. |
| Schandelah | T-OAE | | | | | | <1 to 30 | Non-euxinic (pore-water sulfide) to intermittent euxinia | (25) | | | | |
| Dotternhausen | | | Euxinic | (117) | | | <5 to >80 | Non-euxinic (pore-water sulfide) to intermittent euxinia | (25) | | | | |
| Rietheim | | | | | | | | | | Sedimentological observations: disruption of water mass stratification | (117) | Pyrite, enrichment factors, ratios and co-variation patterns of redox sensitive elements and TOC: anoxic, possibly euxinic | (96) |
| Yorkshire | | | Mostly anoxic to euxinic, occasionally suboxic/hypoxic | (118) | Mo: anoxic and euxinic | (119) | <5 to 60 | Non-euxinic (pore-water sulfide) to intermittent euxinia | (25) | | | | |
| ODP Site 1260 | OAE2 | (97) | Euxinia in black shale intervals | (120) | | | 0 to >100 | Non euxinic to permanent euxinia | (97) | Photic zone euxinia breakdown during cool period | (97) | | |

| | | | | | | | | | | | | |
|---|---|---|---|---|---|---|---|---|---|---|---|---|
| | | Euxinic | (121) | | | | | | Benthic foraminifera: intermittent oxygenation | (121) | | |
| Tarfaya S57 | (81) | Anoxic and periodically euxinic | (122) | | | <5 to >45 | Non-euxinic (pore-water sulfide) to intermittent euxinia | (81) | | | I/Ca and $\delta^{15}N$: regional upwelling of anoxic waters | (123) |
| | (124) | Dominantly euxinic | (124) | | | | | | | | Mo and U EF: anoxic water column | (122) |
| DSDP Site 641 | | | | | | <15 to >6000 | Non euxinic (pore-water sulfide) to permanent euxinia | (37) | | | | |
| DSDP Site 603 | (82) | | | | | ~10 to >45 | Non-euxinic (pore-water sulfide) to intermittent euxinia | (82) | | | | |
| DSDP Site 386 | | | | | | <5 to >65 | Non-euxinic (pore-water sulfide) to intermittent euxinia | (37) | | | | |
| DSDP Site 367 | (82) | | | | | <25 to >300 | Non euxinic (pore-water sulfide) to permanent euxinia | (99) | | | Pyrite enrichment | (40) |
| | | | | | | <25 to ~200 | Non euxinic (pore-water sulfide) to permanent euxinia | (98) | | | | |
| ODP Site 1276 | (125) | | | | | <1 to >20 | Non-euxinic (pore-water sulfide) | (37) | | | Biomarkers: persistent anoxia | (125) |

| | | | | | | | | | | | | | |
|---|---|---|---|---|---|---|---|---|---|---|---|---|---|
| DSDP Site 530 | | (99) | | | | | <10 to >200 | Non euxinic (pore-water sulfide) to permanent euxinia | (99) | Biomarkers: dynamic setting with subtle bottom water reoxygena-tion | (99) | Biomarkers: anoxia and euxinia | (99) |
| Bass River | | | | | | | <2 | Non-euxinic (shale value) | (83) | | | | |
| Wunstorf | | | | | | | <1 | Non-euxinic (shale value) | (100) | Periodic black shale deposition | (100) | | |
| Bass River | | | | | | | <2 | Non-euxinic (shale value) | (57) | | | | |
| IODP Site M006, ACEX | | (127) | | | | | <5 to >100 | Non euxinic (pore-water sulfide) to permanent euxinia | (57) | Organic foraminiferal linings: during termination of event | (104) | Biomarkers: euxinia of variable intensity | (127) |
| IB10 | | (104) | | | | | <10 | Non-euxinic (pore-water sulfide) | (104) | | | | |
| ODP Site 959 | PETM | | | | | | | | | Organic foraminiferal linings: during carbon isotope plateau | (105) | | |
| IODP Site 1403 | | | | | | | <5 | Non-euxinic (pore-water sulfide) | (57) | | | | |
| ODP Site 1172 | | | | | | | <2 | Non-euxinic (shale value) | (57) | | | | |
| ODP Site 752 | | | | | | | <1 | Non-euxinic (shale value) | (57) | | | | |
| Forada | | | | | | | - | Non-euxinic | (57) | | | | |
| Dzhengutay | | | | | | <5 | Non-euxinic (pore-water sulfide) | (28) | | | | | |

| | | | | | | | | | | | | | |
|---|---|---|---|---|---|---|---|---|---|---|---|---|---|
| Guru Fatima | | Possibly anoxic to euxinic | (111) | | | <5 to <100 | Non-euxinic (pore-water sulfide) to intermittent euxinia | (28) | DOP and lycopane fluctuations: fluctuating chemocline and periodic oxygenation of seafloor | (28) | | | |
| Kheu River | | Possibly anoxic to possibly euxinic | (111) | | | <1 to >1000 | Non euxinic (pore-water sulfide) to permanent euxinia | (28) | | | | | |
| ODP Site 969 | i-282c | (30) | | | S isotopes (pyrite): euxinic water column | (30) | <70 to >700 | Intermittent to permanent euxinia | This study ((38)) | | | Pyrite enrichment | (30) |
| PS25PC | S5 | | | | | | <30 to <150 | Intermittent to permanent euxinia | (39) | | | | |
| KC19C | | | | | | | | | | | | $C_{ORG}/P_{ORG}$ vs Sed. rates: low oxygen/anoxic | (39) |
| MS21PC | S1 | | | | | | <1 to >23 | Non-euxinic (pore-water sulfide) | (86) | | | Mo and U EF: suboxic to euxinic | (86) |
| KC19C | | | | | | | | | | | | $C_{ORG}/P_{ORG}$ vs Sed. rates: transitional between oxic and anoxic | (86) |

*: Photic Zone Euxinia

**Supplementary data set DS1.** Previously unpublished $C_{ORG}/P_{TOT}$, Fe/Al and $CaCO_3$ data. The $C_{ORG}/P_{TOT}$ and Fe/Al data are included in main text figures 1 and 2. For further information on these datasets, including references to the original publications, see the Supplementary Text.

**REFERENCES AND NOTES**

1. T. Tyrrell, The relative influences of nitrogen and phosphorus on oceanic primary production. *Nature* **400**, 525–531 (1999).

2. P. Van Cappellen, E. D. Ingall, Redox stabilization of the atmosphere and oceans by phosphorus-limited marine productivity. *Science* **271**, 493–496 (1996).

3. K. C. Ruttenberg, The global phosphorus cycle. *TrGeo* **8**, 585–643 (2003).

4. P. Van Cappellen, E. D. Ingall, Benthic phosphorus regeneration, net primary production, and ocean anoxia: A model of the coupled marine biogeochemical cycles of carbon and phosphorus. *Paleoceanogr. Paleoclimatol.* **9**, 677–692 (1994).

5. T. J. Algeo, E. Ingall, Sedimentary $C_{org}$: P ratios, paleocean ventilation, and Phanerozoic atmospheric $pO_2$. *Palaeogeogr. Palaeoclimatol. Palaeoecol.* **256**, 130–155 (2007).

6. H. E. Hartnett, R. G. Keil, J. I. Hedges, A. H. Devol, Influence of oxygen exposure time on organic carbon preservation in continental margin sediments. *Nature* **391**, 572–575 (1998).

7. E. D. Ingall, R. M. Bustin, P. Van Cappellen, Influence of water column anoxia on the burial and preservation of carbon and phosphorus in marine shales. *Geochim. Cosmochim. Acta* **57**, 303–316 (1993).

8. A. K. Steenbergh, P. L. Bodelier, H. L. Hoogveld, C. P. Slomp, H. J. Laanbroek, Phosphatases relieve carbon limitation of microbial activity in Baltic Sea sediments along a redox-gradient. *Limnol. Oceanogr.* **56**, 2018–2026 (2011).

9. P. Kraal, C. P. Slomp, A. Forster, M. M. M. Kuypers, Phosphorus cycling from the margin to abyssal depths in the proto-Atlantic during Oceanic Anoxic Event 2. *Palaeogeogr. Palaeoclimatol. Palaeoecol.* **295**, 42–54 (2010).

10. S. Flögel, K. Wallmann, C. J. Poulsen, J. Zhou, A. Oschlies, S. Voigt, W. Kuhnt, Simulating the biogeochemical effects of volcanic CO2 degassing on the oxygen-state of the deep ocean during the cenomanian/turonian anoxic event (OAE2). *Earth Planet. Sci. Lett.* **305**, 371–384 (2011).

11. P. Kraal, N. Dijkstra, T. Behrends, C. P. Slomp, Phosphorus burial in sediments of the sulfidic deep Black Sea: Key roles for adsorption by calcium carbonate and apatite authigenesis. *Geochim. Cosmochim. Acta* **204**, 140–158 (2017).

12. R. A. Jahnke, The synthesis and solubility of carbonate fluorapatite. *Am. J. Sci.* **284**, 58–78 (1984).

13. P. Koutsoukos, Z. Amjad, M. B. Tomson, G. H. Nancollas, Crystallization of calcium phosphates. A constant composition study. *J. Am. Chem. Soc.* **102**, 1553–1557 (1980).

14. W. C. Burnett, Geochemistry and origin of phosphorite deposits from off Peru and Chile. *Geol. Soc. Am. Bull.* **88**, 813–823 (1977).

15. H. C. Jenkyns, Geochemistry of oceanic anoxic events. *Geochem. Geophys. Geosyst.* **11**, Q03004 (2010).

16. B. C. Gill, T. W. Lyons, H. C. Jenkyns, A global perturbation to the sulfur cycle during the toarcian oceanic anoxic event. *Earth Planet. Sci. Lett.* **312**, 484–496 (2011).

17. J. D. Owens, B. C. Gill, H. C. Jenkyns, S. M. Bates, S. Severmann, M. M. Kuypers, T. W. Lyons, Sulfur isotopes track the global extent and dynamics of euxinia during Cretaceous Oceanic Anoxic Event 2. *Proc. Natl. Acad. Sci. U.S.A.* **110**, 18407–18412 (2013).

18. A. J. Dickson, H. C. Jenkyns, D. Porcelli, S. van den Boorn, E. Idiz, Basin-scale controls on the molybdenum-isotope composition of seawater during Oceanic Anoxic Event 2 (Late Cretaceous). *Geochim. Cosmochim. Acta* **178**, 291–306 (2016).

19. A. Sluijs, L. van Roij, G. J. Harrington, S. Schouten, J. A. Sessa, L. J. LeVay, C. P. Slomp, Warming, euxinia and sea level rise during the Paleocene-Eocene thermal maximum on the Gulf coastal plain: Implications for ocean oxygenation and nutrient cycling. *Clim. Past* **10**, 1421–1439.

20. W. Yao, A. Paytan, U. G. Wortmann, Large-scale ocean deoxygenation during the Paleocene-Eocene thermal maximum. *Science* **361**, 804–806 (2018).

21. F. A. McInerney, S. L. Wing, The Paleocene-Eocene thermal maximum: A perturbation of carbon cycle, climate, and biosphere with implications for the future. *Annu. Rev. Earth Planet. Sci.* **39**, 489–516 (2011).

22. A. Trecalli, J. Spangenberg, T. Adatte, K. B. Föllmi, M. Parente, Carbonate platform evidence of ocean acidification at the onset of the early Toarcian oceanic anoxic event. *Earth Planet. Sci. Lett.* **357**, 214–225 (2012).

23. R. E. Zeebe, T. Tyrrell, History of carbonate ion concentration over the last 100 million years II: Revised calculations and new data. *Geochim. Cosmochim. Acta* **257**, 373–392 (2019).

24. J. C. Zachos, U. Röhl, S. A. Schellenberg, A. Sluijs, D. A. Hodell, D. C. Kelly, H. McCarren, Rapid acidification of the ocean during the Paleocene-Eocene thermal maximum. *Science* **308**, 1611–1615 (2005).

25. I. R. Baroni, A. Pohl, N. A. van Helmond, N. M. Papadomanolaki, A. L. Coe, A. S. Cohen, C. P. Slomp, Ocean circulation in the Toarcian (Early Jurassic): A key control on deoxygenation and carbon burial on the European shelf. *Paleoceanogr. Paleoclimatol.* **33**, 994–1012 (2018).

26. H. P. Mort, T. Adatte, K. B. Föllmi, G. Keller, P. Steinmann, V. Matera, D. Stüben, Phosphorus and the roles of productivity and nutrient recycling during Oceanic Anoxic Event 2. *Geology* **35**, 483–486 (2007).

27. R. Takashima, H. Nishi, B. T. Huber, R. M. Leckie, Greenhouse world and the Mesozoic ocean. *Oceanography* **19**, 64–74 (2006).

28. A. J. Dickson, R. L. Rees-Owen, C. März, A. L. Coe, A. S. Cohen, R. D. Pancost, E. Shcherbinina, The spread of marine anoxia on the northern Tethys margin during the Paleocene-Eocene thermal maximum. *Paleoceanogr. Paleoclimatol.* **29**, 471–488 (2014).

29. D. J. Beerling, D. L. Royer, Convergent cenozoic $CO_2$ history. *Nat. Geosci.* **4**, 418–420 (2011).

30. H. F. Passier, J. J. Middelburg, G. J. de Lange, M. E. Böttcher, Modes of sapropel formation in the eastern Mediterranean: Some constraints based on pyrite properties. *Mar. Geol.* **153**, 199–219 (1999).

31. E. J. Rohling, G. Marino, K. M. Grant, Mediterranean climate and oceanography, and the periodic development of anoxic events (sapropels). *Earth Sci. Rev.* **143**, 62–97 (2015)

32. P. Kraal, C. P. Slomp, D. C. Reed, G. J. Reichart, S. W. Poulton, Sedimentary phosphorus and iron cycling in and below the oxygen minimum zone of the northern Arabian Sea. *Biogeosciences* **9**, 2603–2624 (2012).

33. R. Raiswell, D. S. Hardisty, T. W. Lyons, D. E. Canfield, J. D. Owens, N. J. Planavsky, C. T. Reinhard, The iron paleoredox proxies: A guide to the pitfalls, problems and proper practice. *Am. J. Sci.* **318**, 491–526 (2018).

34. C. Scott, T. W. Lyons, Contrasting molybdenum cycling and isotopic properties in euxinic versus non-euxinic sediments and sedimentary rocks: Refining the paleoproxies. *Chem. Geol.* **324-325**, 19–27 (2012).

35. A. C. Redfield, *On the Proportions of Organic Derivatives in Sea Water and Their Relation to the Composition of Plankton (Vol. 1)* (University Press of Liverpool, 1934).

36. M. B. Andersen, A. Matthews, M. Bar-Matthews, D. Vance, Rapid onset of ocean anoxia shown by high U and low Mo isotope compositions of sapropel S1. *Geochem. Perspect. Lett.* **15**, 10–14 (2020).

37. N. A. van Helmond, I. R. Baroni, A. Sluijs, J. S. S. Damsté, C. P. Slomp, Spatial extent and degree of oxygen depletion in the deep proto-North Atlantic basin during Oceanic Anoxic Event 2. *Geochem. Geophys. Geosyst.* **15**, 4254–4266 (2014).

38. C. P. Slomp, J. Thomson, G. J. de Lange, Controls on phosphorus regeneration and burial during formation of eastern Mediterranean sapropels. *Mar. Geol.* **203**, 141–159 (2004).

39. P. Kraal, C. P. Slomp, G. J. de Lange, Sedimentary organic carbon to phosphorus ratios as a redox proxy in Quaternary records from the Mediterranean. *Chem. Geol.* **277**, 167–177 (2010).

40. P. Kraal, C. P. Slomp, A. Forster, M. M. Kuypers, A. Sluijs, Pyrite oxidation during sample storage determines phosphorus fractionation in carbonate-poor anoxic sediments. *Geochim. Cosmochim. Acta* **73**, 3277–3290 (2009).

41. M. Zhao, S. Zhang, L. G. Tarhan, C. T. Reinhard, N. Planavsky, The role of calcium in regulating marine phosphorus burial and atmospheric oxygenation. *Nat. Commun.* **11**, 1–8 (2020).

42. K. C. Ruttenberg, R. A. Berner, Authigenic apatite formation and burial in sediments from non-upwelling, continental margin environments. *Geochim. Cosmochim. Acta* **57**, 991–1007 (1993).

43. C. P. Slomp, E. H. Epping, W. Helder, W. V. Raaphorst, A key role for iron-bound phosphorus in authigenic apatite formation in North Atlantic continental platform sediments. *J. Mar. Res.* **54**, 1179–1205 (1996).

44. P. Van Cappellen, R. A. Berner, Fluorapatite crystal growth from modified seawater solutions. *Geochim. Cosmochim. Acta* **55**, 1219–1234 (1991).

45. A. Gunnars, S. Blomqvist, C. Martinsson, Inorganic formation of apatite in brackish seawater from the Baltic Sea: An experimental approach. *Mar. Chem.* **91**, 15–26 (2004).

46. K. Mänd, K. Kirsimäe, A. Lepland, C. H. Crosby, J. V. Bailey, K. O. Konhauser, K. Lumiste, Authigenesis of biomorphic apatite particles from Benguela upwelling zone sediments off Namibia: The role of organic matter in sedimentary apatite nucleation and growth. *Geobiology* **16**, 640–658 (2018).

47. J. Diaz, E. Ingall, C. Benitez-Nelson, D. Paterson, M. D. de Jonge, I. McNulty, J. A. Brandes, Marine polyphosphate: A key player in geologic phosphorus sequestration. *Science* **320**, 652–655 (2008).

48. A. Forster, S. Schouten, K. Moriya, P. A. Wilson, J. S. S. Damsté, Tropical warming and intermittent cooling during the Cenomanian/Turonian Oceanic Anoxic Event 2: Sea surface temperature records from the equatorial Atlantic. *Paleoceanogr. Paleoclimatol.* **22**, 1219 (2007).

49. O. Friedrich, R. D. Norris, J. Erbacher, Evolution of middle to Late Cretaceous oceans—A 55 m.y. record of Earth's temperature and carbon cycle. *Geology* **40**, 107–110 (2012).

50. D. J. Beerling, M. R. Lomas, D. R. Gröcke, On the nature of methane gas-hydrate dissociation during the Toarcian and Aptian oceanic anoxic events. *Am. J. Sci.* **302**, 28–49 (2002).

51. T. R. Bailey, Y. Rosenthal, J. M. McArthur, B. Van de Schootbrugge, M. F. Thirlwall, Paleoceanographic changes of the Late Pliensbachian–Early Toarcian interval: A possible link to the genesis of an Oceanic Anoxic Event. *Earth Planet. Sci. Lett.* **212**, 307–320 (2003).

52. C. V. Ullmann, R. Boyle, L. V. Duarte, S. P. Hesselbo, S. A. Kasemann, T. Klein, M. Aberhan, Warm afterglow from the Toarcian Oceanic Anoxic Event drives the success of deep-adapted brachiopods. *Sci. Rep.* **10**, 1–11 (2020).

53. K. C. Emeis, H. Schulz, U. Struck, M. Rossignol-Strick, H. Erlenkeuser, M. W. Howell, T. Sakamoto, Eastern Mediterranean surface water temperatures and δ18O composition during deposition of sapropels in the late Quaternary. *Paleoceanogr. Paleoclimatol.* **18**, 1005 (2003).

54. G. S. Dwyer, T. M. Cronin, P. A. Baker, M. E. Raymo, J. S. Buzas, T. Corrège, North Atlantic deepwater temperature change during late Pliocene and late Quaternary climatic cycles. *Science* **270**, 1347–1351 (1995).

55. MEDAR Group, Medatlas/2002-database: Mediterranean and Black Sea database of temperature, salinity and bio-chemical parameters. Climatological atlas (2003); http://nettuno.ogs.trieste.it/medar/climatologies/DH3/dh3.html [accessed 2020].

56. C. Shao, Y. Sui, D. Tang, L. Legendre, Spatial variability of surface-sediment porewater pH and related water-column characteristics in deep waters of the northern South China Sea. *Prog. Oceanogr.* **149**, 134–144 (2016).

57. N. M. Papadomanolaki, A. Sluijs, C. P. Slomp, Eutrophication and deoxygenation forcing of marginal marine organic carbon burial during the PETM. *Paleoceanogr. Paleoclimatol.* **37**, e2021PA004232 (2022).

58. E. Erba, F. Tremolada, Nannofossil carbonate fluxes during the Early Cretaceous: Phytoplankton response to nutrification episodes, atmospheric $CO_2$, and anoxia. *Paleoceanogr. Paleoclimatol.* **19**, PA1008 (2004).

59. M. O. Clarkson, C. H. Stirling, H. C. Jenkyns, A. J. Dickson, D. Porcelli, C. M. Moy, T. M. Lenton, Uranium isotope evidence for two episodes of deoxygenation during Oceanic Anoxic Event 2. *Proc. Natl. Acad. Sci. U.S.A.* **115**, 2918–2923 (2018).

60. A. J. Dickson, A molybdenum-isotope perspective on Phanerozoic deoxygenation events. *Nat. Geosci.* **10**, 721–726 (2017).

61. M. O. Clarkson, T. M. Lenton, M. B. Andersen, M. L. Bagard, A. J. Dickson, D. Vance, Upper limits on the extent of seafloor anoxia during the PETM from uranium isotopes. *Nat. Commun.* **12**, 1–9 (2021).

62. I. Jarvis, J. S. Lignum, D. R. Gröcke, H. C. Jenkyns, M. A. Pearce, Black shale deposition, atmospheric $CO_2$ drawdown, and cooling during the cenomanian-turonian oceanic anoxic event. *Paleoceanogr. Paleoclimatol.* **26**, PA3201 (2011).

63. G. J. Bowen, J. C. Zachos, Rapid carbon sequestration at the termination of the Palaeocene–Eocene thermal maximum. *Nat. Geosci.* **3**, 866–869 (2010).

64. E. J. Rohling, Review and new aspects concerning the formation of eastern Mediterranean sapropels. *Mar. Geol.* **122**, 1–28 (1994).

65. V. Palastanga, C. P. Slomp, C. Heinze, Glacial-interglacial variability in ocean oxygen and phosphorus in a global biogeochemical model. *Biogeosciences* **10**, 945–958 (2013).

66. S. Beil, W. Kuhnt, A. Holbourn, F. Scholz, J. Oxmann, K. Wallmann, E. H. Chellai, Cretaceous oceanic anoxic events prolonged by phosphorus cycle feedbacks. *Clim. Past* **16**, 757–782 (2020).

67. M. Schobben, W. J. Foster, A. R. Sleveland, V. Zuchuat, H. H. Svensen, S. Planke, S. W. Poulton, A nutrient control on marine anoxia during the end-Permian mass extinction. *Nat. Geosci.* **13**, 640–646 (2020).

68. R. T. Wilkin, M. A. Arthur, W. E. Dean, History of water-column anoxia in the Black Sea indicated by pyrite framboid size distributions. *Earth Planet. Sci. Lett.* **148**, 517–525 (1997).

69. L. D. Anderson, M. L. Delaney, K. L. Faul, Carbon to phosphorus ratios in sediments: Implications for nutrient cycling. *Global Biogeochem. Cycles* **15**, 65–79 (2001).

70. C. P. Slomp, J. Thomson, G. J. de Lange, Enhanced regeneration of phosphorus during formation of the most recent eastern Mediterranean sapropel (S1). *Geochim. Cosmochim. Acta* **66**, 1171–1184 (2002).

71. L. M. Eijsink, M. D. Krom, B. Herut, Speciation and burial flux of phosphorus in the surface sediments of the eastern Mediterranean. *Am. J. Sci.* **300**, 483–503 (2000).

72. J. M. McArthur, T. J. Algeo, B. Van de Schootbrugge, Q. Li, R. J. Howarth, Basinal restriction, black shales, Re-Os dating, and the Early Toarcian (Jurassic) oceanic anoxic event. *Paleoceanogr. Paleoclimatol.* **23**, PA4217 (2008).

73. T. J. Algeo, T. W. Lyons, Mo–Total organic carbon covariation in modern anoxic marine environments: Implications for analysis of paleoredox and paleohydrographic conditions. *Paleoceanogr. Paleoclimatol.* **21**, PA1016 (2006).

74. W. K. Lenstra, M. Egger, N. A. Van Helmond, E. Kritzberg, D. J. Conley, C. P. Slomp, Large variations in iron input to an oligotrophic Baltic Sea estuary: Impact on sedimentary phosphorus burial. *Biogeosciences* **15**, 6979–6996 (2018).

75. Y. Wang, P. A. Van A multicomponent reactive transport model of early diagenesis: Application to redox cycling in coastal marine sediments. *Geochim. Cosmochim. Acta* **60**, 2993–3014 (1996).

76. P. Froelich, G. P. Klinkhammer, M. L. Bender, N. A. Luedtke, G. R. Heath, D. Cullen, V. Maynard, Early oxidation of organic matter in pelagic sediments of the eastern equatorial Atlantic: Suboxic diagenesis. *Geochim. Cosmochim. Acta* **43**, 1075–1090 (1979).

77. B. P. Boudreau, *Diagenetic Models and Their Implementation (Vol. 410)* (Springer, 1997).

78. D. L. Parkhurst, C. A. J. Appelo, Description of input and examples for PHREEQC version 3: A computer program for speciation, batch-reaction, one-dimensional transport, and inverse geochemical calculations (No. 6-A43, U.S. Geological Survey, 2013).

79. C. A. J. Appelo, D. Postma, *Geochemistry, Groundwater and Pollution* (CRC Press, 2004).

80. A. Grothe, F. Andreetto, G. J. Reichart, M. Wolthers, C. G. Van Baak, I. Vasiliev, W. Krijgsman, Paratethys pacing of the Messinian salinity crisis: Low salinity waters contributing to gypsum precipitation? *Earth Planet. Sci. Lett.* **532** 116029 (2020).

81. S. Kolonic, T. Wagner, A. Forster, J. S. S. Damsté, B. Walsworth-Bell, E. Erba, M. M. Kuypers, Black shale deposition on the northwest African Shelf during the Cenomanian/Turonian oceanic anoxic event: Climate coupling and global organic carbon burial. *Paleoceanogr. Paleoclimatol.* **20**, PA1006 (2005).

82. M. M. Kuypers, L. J. Lourens, W. I. C. Rijpstra, R. D. Pancost, I. A. Nijenhuis, J. S. S. Damsté, Orbital forcing of organic carbon burial in the proto-North Atlantic during Oceanic Anoxic Event 2. *Earth Planet. Sci. Lett.* **228**, 465–482 (2004).

83. N. A. van Helmond, A. Sluijs, G. J. Reichart, J. S. S. Damsté, C. P. Slomp, H. Brinkhuis, A perturbed hydrological cycle during Oceanic Anoxic Event 2. *Geology* **42**, 123–126 (2014).

84. N. A. G. M. Van Helmond, A. Sluijs, J. S. S. Damsté, G. J. Reichart, S. Voigt, J. Erbacher, H. Brinkhuis, Freshwater discharge controlled deposition of Cenomanian–Turonian black shales on the NW European epicontinental shelf (Wunstorf, northern Germany). *Clim. Past* **11** 495–508 (2015).

85. H. F. Passier, M. J. Dekkers, G. J. de Lange, Sediment chemistry and magnetic properties in an anomalously reducing core from the eastern Mediterranean Sea. *Chem. Geol.* **152**, 287–306 (1998).

86. K. L. Zwiep, R. Hennekam, T. H. Donders, N. A. Van Helmond, G. J. De Lange, F. Sangiorgi, Marine productivity, water column processes and seafloor anoxia in relation to Nile discharge during sapropels S1 and S3. *Quat. Sci. Rev.* **200**, 178–190 (2018).

87. C. Montoya-Pino, S. Weyer, A. D. Anbar, J. Pross, W. Oschmann, B. van de Schootbrugge, H. W. Arz, Global enhancement of ocean anoxia during Oceanic Anoxic Event 2: A quantitative approach using U isotopes. *Geology* **38**, 315–318 (2010).

88. F. M. Monteiro, R. D. Pancost, A. Ridgwell, Y. Donnadieu, Nutrients as the dominant control on the spread of anoxia and euxinia across the Cenomanian-Turonian oceanic anoxic event (OAE2): Model-data comparison. *Paleoceanogr. Paleoclimatol.* **27**, PA4209 (2012).

89. D. C. Reed, C. P. Slomp, G. J. de Lange, A quantitative reconstruction of organic matter and nutrient diagenesis in Mediterranean Sea sediments over the Holocene. *Geochim. Cosmochim. Acta* **75**, 5540–5558 (2011).

90. B. J. Haupt, D. Seidov, Warm deep-water ocean conveyor during Cretaceous time. *Geology* **29**, 295–298 (2001).

91. O. Friedrich, J. Erbacher, K. Moriya, P. A. Wilson, H. Kuhnert, Warm saline intermediate waters in the Cretaceous tropical Atlantic Ocean. *Nat. Geosci.* **1**, 453–457 (2008).

92. A. Tripati, H. Elderfield, Deep-sea temperature and circulation changes at the Paleocene-Eocene thermal maximum. *Science* **308**, 1894–1898 (2005).

93. T. D. Jones, D. J. Lunt, D. N. Schmidt, A. Ridgwell, A Sluijs, P. J. Valdes, M. Maslin, Climate model and proxy data constraints on ocean warming across the Paleocene–Eocene Thermal Maximum. *Earth Sci. Rev.* **125**, 123–145 (2013).

94. K. M. Grant, R. Grimm, U. Mikolajewicz, G. Marino, M. Ziegler, E. J. Rohling, The timing of Mediterranean sapropel deposition relative to insolation, sea-level and African monsoon changes. *Quat. Sci. Rev.* **140**, 125–141 (2016).

95. S. M. Harding, "The Toarcian Oceanic Anoxic Event: Organic and inorganic geochemical anomalies in organic-carbon-rich mudrocks from the North Yorkshire coast, UK and Dotternhausen Quarry, SW Germany," thesis, The Open University, UK (2004).

96. J. C. Montero-Serrano, K. B. Föllmi, T. Adatte, J. E. Spangenberg, N. Tribovillard, A. Fantasia, G. Suan, Continental weathering and redox conditions during the early Toarcian oceanic anoxic event in the northwestern Tethys: Insight from the Posidonia Shale section in the Swiss Jura Mountains. *Palaeogeogr. Palaeoclimatol. Palaeoecol.* **429**, 83–99 (2015).

97. E. C. van Bentum, A. Hetzel, H. J. Brumsack, A. Forster, G. J. Reichart, J. S. S. Damsté, Reconstruction of water column anoxia in the equatorial Atlantic during the Cenomanian–Turonian oceanic anoxic event using biomarker and trace metal proxies. *Palaeogeogr. Palaeoclimatol. Palaeoecol.* **280**, 489–498 (2009).

98. J. D. Owens, T. W. Lyons, X. Li, K. G. Macleod, G. Gordon, M. M. Kuypers, S. Severmann, Iron isotope and trace metal records of iron cycling in the proto-North Atlantic during the Cenomanian-Turonian oceanic anoxic event (OAE-2). *Paleoceanogr. Paleoclimatol.* **27**, PA3223 (2012).

99. M. M. Kuypers, R. D. Pancost, I. A. Nijenhuis, J. S. Sinninghe Damsté, Enhanced productivity led to increased organic carbon burial in the euxinic North Atlantic basin during the late Cenomanian oceanic anoxic event. *Paleoceanogr. Paleoclimatol.* **17**, 3-1–3-13 (2002).

100. A. Forster, M. M. Kuypers, S. C. Turgeon, H. J. Brumsack, M. R. Petrizzo, J. S. S. Damsté, The Cenomanian/Turonian oceanic anoxic event in the South Atlantic: New insights from a geochemical study of DSDP Site 530A. *Palaeogeogr. Palaeoclimatol. Palaeoecol.* **267**, 256–283 (2008).

101. A. Hetzel, C. März, C. Vogt, H. J. Brumsack, Geochemical environment of Cenomanian-Turonian black shale deposition at Wunstorf (northern Germany). *Cretaceous Res.* **32**, 480–494 (2011).

102. C. M. John, S. M. Bohaty, J. C. Zachos, A. Sluijs, S. Gibbs, H. Brinkhuis, T. J. Bralower, North American continental margin records of the Paleocene-Eocene thermal maximum: Implications for global carbon and hydrological cycling. *Paleoceanogr. Paleoclimatol.* **23**, PA2217 (2008).

103. A. Sluijs, U. Röhl, S. Schouten, H. J. Brumsack, F. Sangiorgi, J. S. S. Damsté, H. Brinkhuis, Arctic late Paleocene–early Eocene paleoenvironments with special emphasis on the Paleocene-Eocene thermal maximum (Lomonosov Ridge, Integrated Ocean Drilling Program Expedition 302). *Paleoceanogr. Paleoclimatol.* **23**, PA1S11 (2008).

104. J. Frieling, H. Gebhardt, M. Huber, O. A. Adekeye, S. O. Akande, G. J. Reichart, A. Sluijs, Extreme warmth and heat-stressed plankton in the tropics during the Paleocene-Eocene thermal maximum. *Sci. Adv.* **3**, e1600891 (2017).

105. J. Frieling, G. J. Reichart, J. J. Middelburg, U. Röhl, T. Westerhold, S. M. Bohaty, A. Sluijs, Tropical atlantic climate and ecosystem regime shifts during the Paleocene–Eocene thermal maximum. *Clim. Past* **14**, 39–55 (2018).

106. D. E. Penman, S. K. Turner, P. F. Sexton, R. D. Norris, A. J. Dickson, S. Boulila, A. Ridgwell, R. E. Zeebe, J. C. Zachos, A. Cameron, T. Westerhold, U. Röhl, (2016). An abyssal carbonate compensation depth overshoot in the aftermath of the Palaeocene–Eocene Thermal Maximum. *Nat. Geosci.* **9**, 575–580.

107. N. M. Papadomanolaki, A. Sluijs, C. P. Slomp, Carbonate geochemistry of ODP Hole 189-1172D. PANGAEA (2021); https://doi.pangaea.de/10.1594/PANGAEA.929307.

108. N. M. Papadomanolaki, A. Sluijs, C. P. Slomp, Carbonate geochemistry of ODP Hole 121-752A. PANGAEA (2021); https://doi.pangaea.de/10.1594/PANGAEA.929304.

109. L. Giusberti, D. Rio, C. Agnini, J. Backman, E. Fornaciari, F. Tateo, M. Oddone, Mode and tempo of the Paleocene-Eocene thermal maximum in an expanded section from the Venetian pre-Alps. *Geol. Soc. Am. Bull.* **119**, 391–412 (2007).

110. N. Komar, R. E. Zeebe, Redox-controlled carbon and phosphorus burial: A mechanism for enhanced organic carbon sequestration during the PETM. *Earth Planet. Sci. Lett.* **479**, 71–82 (2017).

111. T. D. Herbert, G. Ng, L. C. Peterson, Evolution of Mediterranean sea surface temperatures 3.5–1.5 Ma: Regional and hemispheric influences. *Earth Planet. Sci. Lett.* **409**, 307–318 (2015).

112. F. Sangiorgi, E. Dinelli, P. Maffioli, L. Capotondi, S. Giunta, C. Morigi, M. S. Principato, A. Negri, K. C. Emeis, C. Corselli, Geochemical and micropaleontological characterisation of a Mediterranean sapropel S5: A case study from core BAN89GC09 (south of Crete). *Palaeogeogr. Palaeoclimatol. Palaeoecol.* **235**, 192–207 (2006).

113. S. M. Techtmann, J. L. Fortney, K. A. Ayers, D. C. Joyner, T. D. Linley, S. M. Pfiffner, T. C. Hazen, The unique chemistry of Eastern Mediterranean water masses selects for distinct microbial communities by depth. *PLOS ONE* **10**, e0120605 (2015).

114. A. M. Abdel-Halim, M. A. Aly-Eldeen, Characteristics of Mediterranean Sea water in vicinity of Sidikerir Region, west of Alexandria, Egypt. *J. Aquat. Res.* **42**, 133–140 (2016).

115. W. K. Lenstra, M. J. M. Séguret, T. Behrends, R. K. Groeneveld, M. Hermans, R. Witbaard, C. P. Slomp, Controls on the shuttling of manganese over the northwestern Black Sea shelf and its fate in the euxinic deep basin. *Geochim. Cosmochim. Acta* **273**, 177–204 (2020).

116. T. R. Them, Thallium isotopes reveal protracted anoxia during the Toarcian (Early Jurassic) associated with volcanism, carbon burial, and mass extinction. *Proc. Natl. Acad. Sci. U.S.A.* **115**, 6596–6601 (2018).

117. A. Fantasia, K. B. Föllmi, T. Adatte, J. E. Spangenberg, J. C. Montero-Serrano, The Early Toarcian oceanic anoxic event: Paleoenvironmental and paleoclimatic change across the Alpine Tethys (Switzerland). *Global Planet. Change* **162**, 53–68 (2018).

118. N. Thibault, M. Ruhl, C. V. Ullmann, C. Korte, D. B. Kemp, D. R. Gröcke, S. P. Hesselbo, The wider context of the Lower Jurassic Toarcian oceanic anoxic event in Yorkshire coastal outcrops, UK. *Proc. Geol. Assoc.* **129**, 372–391 (2018).

119. C. R. Pearce, A. S. Cohen, A. L. Coe, K. W. Burton, Molybdenum isotope evidence for global ocean anoxia coupled with perturbations to the carbon cycle during the Early Jurassic. *Geology* **36**, 231–234 (2008).

120. M. E. Böttcher, A. Hetzel, H.-J. Brumsack, A. Schipper, Sulfur-iron-carbon geochemistry in sediments of the Demerara Rise. *Proc. Ocean Drill. Progr. Sci. Res.* **207**, 1–23 (2006).

121. A. Hetzel, M. E. Böttcher, U. G. Wortmann, H. J. Brumsack, Paleo-redox conditions during OAE 2 reflected in Demerara Rise sediment geochemistry (ODP Leg 207). *Palaeogeogr. Palaeoclimatol. Palaeoecol.***273**, 302–328 (2009).

122. T. Goldberg, S. W. Poulton, T. Wagner, S. F. Kolonic, M. Rehkämper, Molybdenum drawdown during Cretaceous Oceanic Anoxic Event 2. *Earth Planet. Sci. Lett.* **440**, 81–91 (2016).

123. X. Zhou, H. C. Jenkyns, J. D. Owens, C. K. Junium, X. Y. Zheng, B. B. Sageman, D. S. Hardisty, T. W. Lyons, A. Ridgwell, Z. Lu, Upper ocean oxygenation dynamics from I/Ca ratios during the cenomanian-turonian OAE 2. *Paleoceanogr. Paleoclimatol.* **30**, 510–526 (2015).

124. S. W. Poulton, S. Henkel, C. März, H. Urquhart, S. Flögel, S. Kasten, J. S. S. Damsté, T. Wagner, A continental-weathering control on orbitally driven redox-nutrient cycling during Cretaceous Oceanic Anoxic Event 2. *Geology* **43**, 963–966 (2015).

125. E. C. van Bentum, G. J. Reichart, J. S. S. Damsté, Organic matter provenance, palaeoproductivity and bottom water anoxia during the Cenomanian/Turonian oceanic anoxic event in the Newfoundland Basin (northern proto North Atlantic Ocean). *Org. Geochem.* **50**, 11–18 (2012).

126. A. Sluijs, S. Schouten, M. Pagani, M. Woltering, H. Brinkhuis, J. S. S. Damsté, G. R. Dickens, M. Huber, Gert-Jan Reichart, R. Stein, J. Matthiessen, L. J. Lourens, N. Pedentchouk, J. Backman, K. Moran; The Expedition Scientists, Subtropical Arctic Ocean temperatures during the Palaeocene/Eocene thermal maximum. *Nature* **441**, 610–613 (2006).

127. P. Weller, R. Stein, Paleogene biomarker records from the central Arctic Ocean (Integrated Ocean Drilling Program Expedition 302): Organic carbon sources, anoxia, and sea surface temperature. *Paleoceanogr. Paleoclimatol.* **23**, PA1S17 (2008).
